# Convergent evolution and multi-wave clonal invasion in H3 K27-altered diffuse midline gliomas treated with a PDGFR inhibitor

**DOI:** 10.1186/s40478-022-01381-0

**Published:** 2022-05-31

**Authors:** Sasi Arunachalam, Karol Szlachta, Samuel W. Brady, Xiaotu Ma, Bensheng Ju, Bridget Shaner, Heather L. Mulder, John Easton, Benjamin J. Raphael, Matthew Myers, Christopher Tinkle, Sariah J. Allen, Brent A. Orr, Cynthia J. Wetmore, Suzanne J. Baker, Jinghui Zhang

**Affiliations:** 1 Department of Computational Biology, St. Jude Children’s Research Hospital, MS 1135, Room IA6038, 262 Danny Thomas Place, Memphis, TN 38105 USA; 2 Department of Computer Science, Princeton University, Princeton, NJ 08540 USA; 3 Departments of Radiation Oncology, St. Jude Children’s Research Hospital, Memphis, TN USA; 4 Thermofisher, Waltham, MA 02451 USA; 5 Departments of Pathology, St. Jude Children’s Research Hospital, Memphis, TN USA; 6 Clinical Development, Neoleukin, 188 East Blaine, Suite 450, Seattle, WA 98102 USA; 7 Department of Developmental Neurobiology, St. Jude Children’s Research Hospital, Room M3412, 262 Danny Thomas Place, Memphis, TN 38105 USA

## Abstract

The majority of diffuse midline gliomas, H3 K27-altered (DMG-H3 K27-a), are infiltrating pediatric brain tumors that arise in the pons with no effective treatment. To understand how clonal evolution contributes to the tumor’s invasive spread, we performed exome sequencing and SNP array profiling on 49 multi-region autopsy samples from 11 patients with pontine DMG-H3 K27-a enrolled in a phase I clinical trial of PDGFR inhibitor crenolanib. For each patient, a phylogenetic tree was constructed by testing multiple possible clonal evolution models to select the one consistent with somatic mutations and copy number variations across all tumor regions. The tree was then used to deconvolute subclonal composition and prevalence at each tumor region to study convergent evolution and invasion patterns. Somatic variants in the *PI3K* pathway, a late event, are enriched in our cohort, affecting 70% of patients. Convergent evolution of *PI3K* at distinct phylogenetic branches was detected in 40% of the patients. 24 (~ 50%) of tumor regions were occupied by subclones of mixed lineages with varying molecular ages, indicating multiple waves of invasion across the pons and extrapontine. Subclones harboring a *PDGFRA* amplicon, including one that amplified a *PDGRFA*^Y849C^ mutant allele, were detected in four patients; their presence in extrapontine tumor and normal brain samples imply their involvement in extrapontine invasion. Our study expands the current knowledge on tumor invasion patterns in DMG-H3 K27-a, which may inform the design of future clinical trials.

**Supplementary Information:**

The online version contains supplementary material available at 10.1186/s40478-022-01381-0.

## Introduction

Diffuse midline glioma, H3 K27-altered (DMG-H3 K27-a) is a newly recognized pediatric tumor entity in the fifth edition of WHO Classification of Tumors of the Central Nervous System that includes the majority of brainstem tumors previously termed diffuse intrinsic pontine glioma [1]. DMG-H3 K27-a is a lethal disease with a median overall survival of approximately 10 months [2]. DMG-H3 K27-a accounts for nearly 10–15% of all pediatric brain tumors and 60–80% of all pediatric brain stem gliomas [3, 4]. Surgical intervention is not possible due to the infiltrative nature of the tumor. Diagnostic biopsies are controversial and, at the time this study was initiated, were rarely performed due to the anatomical location of the tumor [5]. Radiotherapy remains the only standard treatment, improving life expectancy by several months [6]. While over 200 clinical trials have been conducted using chemotherapy, none has proven effective in improving life expectancy [5].

Genomic profiling of tumor samples collected from individual or multiple tumor regions of DMG-H3 K27-a patients has identified major genetic drivers. The most prevalent and earliest event is the acquisition of somatic K27M mutation of histone H3 proteins [7–9] most commonly encoded by *H3F3A* and *HIST1H3B*. Mutations in *TP53*, *PPM1D* and *ACVR1* are also reported to be present from diagnosis to end-stage disease, while those affecting the PI3K pathway are late and often subclonal events [7, 8, 10–12].

Aberrant activation of platelet-derived growth factor receptor (PDGFR) signaling is another common event with 20–30% prevalence. Activation occurs via gene amplification or acquisition of point mutations in subclones of DMG-H3 K27-a, and has been directly implicated in driving pediatric glioma formation in vivo [13–16]. Despite the known intratumoral heterogeneity of its somatic alterations, *PDGFRA* is considered a therapeutic target based on abated growth upon treatment with PDGFRA inhibitors and reduced viability after genetic knockout of *PDGFRA* in models of DMG-H3 K27-a glioma [14, 16]. In a recently completed phase I clinical trial of crenolanib, a selective inhibitor of PDGFR activity, no significant difference in overall survival was observed in comparison to historical controls in pediatric patients with newly diagnosed DMG-H3 K27-a or recurrent DMG-H3 K27-a [17]. To gain insight into convergent evolution and patterns of tumor cell invasion, we performed whole exome sequencing (WES) followed by ultra-deep sequencing and SNP array profiling on 49 tumor samples acquired at autopsy from 11 patients enrolled in this trial. Our interest in studying the invasion pattern was motivated in part by a serendipitous finding: while examining tumor-in-normal contamination in DMG-H3 K27-a as part of a clinical pilot study, we discovered an invasive subclone in a histologically normal brain sample distant from the primary tumor [18]. Consequently, we profiled multiple histologically normal brain samples from the same patient in this study so that these samples could serve the dual purpose of providing normal controls for somatic variant analysis and mapping rare subclones involved in extrapontine invasion. These rare subclones can be identified by employing our newly-developed method for in-silico error suppression in deep sequencing data [19].

Mapping of subclonal composition and prevalence at each tumor region is a prerequisite for understanding tumor cell invasion patterns. Such analysis has not been carried out systematically in published multi-region tumor studies including those of DMG-H3 K27-a [20–25]. As a result, there is limited knowledge about subclonal population admixture, which can be caused by migration of tumor cells descending from different phylogenetic branches. To overcome this limitation, we developed a new approach which tests multiple possible clonal evolution models, selecting the one that is consistent with somatic variants (including SNVs/indels and CNVs) across all tumor regions. The resulting data not only showed interesting patterns of convergent evolution, but also revealed co-occupancy of tumor cells descending from different evolution branches in ~ 50% of the tumor regions. The admixed subclonal populations were found in both pontine and extrapontine tumor samples, and intriguingly, in rare tumor populations detected in histologically normal brain tissues. These results indicate multiple waves of DMG-H3 K27-a invasion within the pons and across multiple extrapontine regions. This process of continued clonal evolution and invasion poses a considerable challenge to therapeutic advancement which must be considered during the design of targeted therapy for this deadly disease.

## Materials and methods

### Patient samples and clinical information

**Fig. 1:**
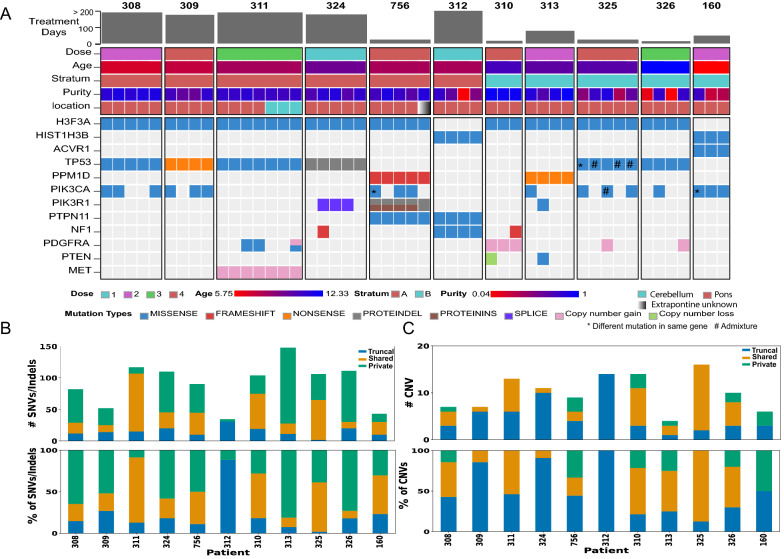
Somatic alterations in multi-region tumor samples acquired from DIPG patients treated with a PDGFR inhibitor. **A** Somatic alterations affecting known DIPG driver genes in 49 tumor samples from 11 DIPG patients treated with crenolanib under stratum A (for newly diagnosed DIPG) or stratum B (for progressive DIPG). Each column represents a single tumor sample from a tumor region in the numeric order of A1, A2, etc.; tumor samples from the same patient are grouped together and labeled by patient ID at the top. Patient and sample metadata are shown at the top while somatic alterations colored by their variant type are shown at the bottom. Within the same patient, presence of a different mutation in the same gene is marked by an asterisk “*” while admixture of multiple mutations in the same sample is marked by “#”. Treatment duration varies broadly ranging from 15 to 658 days. **B** Somatic SNV/indel burden of each patient stratified by their presence in multi-region tumor samples including truncal (detected in all samples), shared (detected in multiple but not all samples) and private mutations (detected in a single sample) shown as the count (top panel) and percentage (bottom panel) of mutations in each patient. **C** Somatic arm-level or whole-chromosome CNVs of each patient shown in the same style as **B**. Only samples profiled from SNP arrays are included and BAF-adjusted CNVs were used for plotting

Eleven patients with clinical and radiologic diagnosis of diffuse intrinsic pontine glioma with H3 K27M mutation, now classified as H3 K27-altered diffuse midline glioma (DMG-H3 K27-a), were enrolled in a clinical trial of the PDGFR inhibitor crenolanib and analyzed for this study. Median age at diagnosis was approximately 8 years (range 5.7–12.3 years). 49 tumor samples and 19 normal samples were acquired by autopsy and subjected to genomic analysis (Fig. 1). Demographical and histopathological characteristics of all specimens are presented in Additional file 3: Table S1. The tumor sample positions were based on the fractional location (e.g. 0.5 corresponds to 6-clock position) within the transverse plane of the pons as reported by the pathologist performing the autopsy. The study was approved by the Institutional Review Board of St. Jude Children’s Research Hospital (protocol XPD-09-018) using patient samples banked with informed consent.

### Somatic variant analysis

WES at ~ 100X coverage for tumor and normal samples was performed as described previously [26] and the sequencing data were aligned to GRCh37-lite with BWA [27]. Somatic SNVs and insertion/deletion (indels) were first identified by Bambino [28] followed by a postprocessing process to filter alignment artifacts, sequencing errors, and paralogs as previously described [29]. We were able to design targeted capture for 78% of curated somatic variants which were subjected to capture-based deep sequencing on tumor and normal samples at the average coverage of > 2500X and the overall validation rate was ~ 94%. To improve the accuracy of detection of mutations with very low variant allele fraction (VAF), we performed computational error suppression using CleanDeepSeq [19] to remove reads likely containing sequencing errors. The cleaned BAM files were used to calculate reference and the mutant allele read counts for targeted SNVs and indels. The indel read counts were based on indelPost, which improves indel allele counts by harmonizing ambiguities via realignment and read-based phasing [30].

### Copy number analysis

We profiled 45 tumors and 11 normal samples by Affymetrix SNP 6.0 array, and somatic copy number variations (CNVs) were derived by incorporating optimal reference normalization and circular binary segmentation [31]. For the four tumor samples that lacked SNP array data (i.e. 326_A1, 324_A1, 325_A1 and 756_A5), we ran CNVkit [32] on paired tumor-normal WES to obtain somatic CNVs.

With the exception of bi-allelic duplication, somatic CNV or copy-neutral loss-of-heterozygosity (LOH) causes a deviation of B-allele fractions (BAFs) of germline heterozygous single nucleotide polymorphisms (SNPs) from the expected BAF of 0.5 in autosomes. The extent of the deviation, referred to as allelic imbalance (AI), is dependent on the cellular prevalence (CP, representing the fraction of all cells—whether tumor or normal—in the sample with the somatic alteration) of the CNV. We used AI to calibrate CPs of somatic CNVs as shown in the example presented in Additional file 2: Fig. S1; details are described in Additional file 1: Supplementary Methods.

### A general model for intra-tumor heterogeneity

Our analysis is built on a core concept of cellular composition of a sample profiled by a molecular assay (e.g. genomic sequencing or SNP array). A tumor sample is comprised of tumor cells and normal cells with their fractions defined as *t* (which corresponds to tumor purity) and *1*−*t*, respectively. Tumor cells are heterogeneous as somatically acquired variants, including copy number variation and sequence mutations, are not always present in all tumor cells. At the sample level BAF-adjusted copy number variation (CNV) is a composite value contributed by the diploid normal cells (*1*−*t* fraction of diploid cells) and tumor cells (*t* fraction) with CNV (*f*) or without CNV (*1*−*f*). Similarly, variant allele fraction (VAF) of a somatic mutation, under a scenario of being acquired after CNV, is affected by the same set of variables as well as the fraction (*g*) of CNV cells that harbor the mutant allele. We used this general model as a starting point for modeling intratumor heterogeneity and clonal evolution presented in subsequent sections.

### Tumor purity estimation

Tumor purity (*t*) can be determined by the clonality of a founder mutation which is expected to be present in every cancer cell. In this study, we used the clonality of *H3F3A* or *HIST1H3B* K27M mutations for tumor purity estimation as these are the known founder mutations in DMG-H3 K27-a [9]. We also examined the cancer cell fraction (CCF) of truncal mutations and CNVs which are present in all tumor regions to ensure the absence of a “superclone” (i.e. having CCF > 1) due to underestimation of tumor purity [33]. An example is presented in Additional file 2: Fig. S2 and details are described in Additional file 1: Supplementary Methods.

To compare our founder mutation-based tumor purity estimate with alternative approaches, we ran two publicly available algorithms, ASCAT and ABSOLUTE. Germline SNPs were based on whole-exome analysis and ASCAT was run using the default setting [34]. We ran ABSOLUTE [24] following the online instructions (https://software.broadinstitute.org/cancer/cga/absolute_run). Copy number segments greater than 1 MB were included to reduce noise and to avoid an out of bounds error when the input data generate excessive segments.

### Using allelic imbalance to model CNV evolution

Although most CNVs can be assigned to a phylogenetic tree based on their presence or absence in the multi-region tumors profiled in each case, a few required focused analysis to evaluate different models. This process is illustrated through the modeling of chr17p loss, which often accompanies *TP53* mutation to cause bi-allelic inactivation of *TP53*. An example is case 325 which is shown in Additional file 2: Fig. S3, details are described in Additional file 1: Supplementary Methods.

### Calculating cancer cell fractions of somatic mutation clusters and CNVs

Under the general model, copy number variation (*c*) in an autosome is a composite value contributed by the diploid normal cells (*1*−*t* fraction of diploid cells) and tumor cells (*t* fraction) with or without CNV:

$$c = \left( {1 - t} \right)2 + t\left[ {\left( {1 - f} \right)2 + fM} \right]$$

where *M* is the integer representing CNV state (*M ∊ {0,1,3,…}*) and *f* is the CCF of tumor cells with a CNV, defined as follows:

$$f = \frac{c - 2}{{\left( {M - 2} \right)t}}$$

Similarly, the VAF ($v$) of a somatic mutation acquired in $g$ fraction of CNV-positive tumor cells, is determined by tumor purity (*t*), fraction of tumor cells with CNV (*f*), fraction of CNV-cells with the mutation (*g*), and multiplicity of the mutant allele (*n*) normalized by the regional copy number state as follows:

$$v = tfgn/c$$

And this leads to:

$$g = vc/tfn$$

Note the following condition: *f,g ∊*  < *0;1* > must be satisfied or the model will be rendered incorrect and an alternative model should be considered. In this analysis, we assume that one mutation belongs to a single lineage, as re-mutation must be extremely rare given a median background mutation rate of 7 × 10^−7^ per base pair in this cohort. Consequently, we require that a correct model be consistent with empirical data in all tumor regions that harbor the mutation.

The CCF of a mutation (SNV or indel) can be derived subsequently as follows:

$$CCF = gf$$

An example of calculating CCF of a *PDGFRA* amplicon harboring PDGFRA^Y849C^ is presented in Additional file 2: Fig. S4 and the details are described in Additional file 1: Supplementary Methods.

### Construction of phylogenetic trees and clonal decomposition for each tumor sample

For each patient, somatic mutations across all tumor regions were clustered by running PyClone [35] using VAFs of SNVs and indels derived from deep sequencing after error suppression, BAF-adjusted somatic CNVs and tumor purity derived from histone H3 K27M mutations. Phylogenetic trees were constructed using the CCF of mutation clusters following two principles described previously, namely the sum’s rule (or the pigeonhole principle) to account for CCFs of mutation clusters in each tumor, and the crossing rule to account for CCFs across all tumor samples [25, 36]. For a mutation cluster detected in multiple tumor regions, the tumor sample likely to produce the most accurate measure of CCF (high tumor purity and high VAF) was prioritized for lineage construction. Additional modeling was performed to further refine the phylogenetic trees as described in Additional file 1: Supplementary Methods.

Clonal composition of each tumor region was mapped based on the CCFs of mutational clusters on each node of the phylogenetic tree. A founder clone consisting only of truncal variants was added if the summed clonal composition derived from its descending nodes was < 1.0. It should be noted that under this circumstance a founder clone can also represent a rare descendant subclone whose lineage-specific mutations were not detectable by WES.

The tumor samples with low purity (< 5%) were not included for phylogenetic tree construction. Their clonal compositions were inferred from matching mutation clusters determined from high purity samples, which can lead to an underestimate of the real clonal diversity in these regions.

### Assessment of residual tumor content in normal brain samples

To determine whether anatomically-normal brain samples contained residual tumor, we first assessed the presence of the founder mutation *H3F3A* K27M using the deep sequencing data generated from normal samples of the 9 patients that had *H3F3A* K27M. This was followed by performing FDR tests of the founder mutation and the relevant mutation clusters. The details are described in Additional file 1: Supplementary Methods and the results are presented in Additional file 2: Fig. S5. 

#### Directional allelic imbalance in CNV

The maternal/paternal allele imbalance was calculated by The REpeat Chromosomal changes Uncovered by Reflection (RECUR) software which compares BAF shifts across multiple tumor samples in two possible directions, reflecting an increased or decreased abundance of the maternal haplotype, relative to the paternal [37]. Tumor BAFs with increased or decreased abundance of the maternal haplotype (relative to the paternal haplotype) were visualized using a two-color scheme and by a shift in the directions of the expected BAF values [37].

#### Statistical analysis

Statistical analysis was carried out using R (www.r-project.org) [38] and python with scipy, numpy, and pandas libraries from https://numpy.org/ and python data analysis library (pydata.org) [39].

## Results

### Mutational profile of DMG-H3 K27-a patients treated with PDGFR inhibitor

A total of 68 autopsy samples, 49 tumor (45 pons and 4 extrapontine) and 19 normal, were obtained from eleven patients; a median of 4 tumor samples (range 3–7) per patient was analyzed. The patient cohort was comprised of 6 newly diagnosed and 5 progressive DMG-H3 K27-a patients enrolled under stratum A and B, respectively, in a phase I clinical trial investigating the efficacy of crenolanib (Fig. 1A, Additional file 3: Table S1). The median treatment duration is 79 days (range 15–658 days), and approximately half (55%) of the patients had a relatively short treatment duration of < 100 days. A total of 1,359 somatic SNVs and indels were identified from paired tumor-normal WES data, 80% of which had their variant allele fraction (VAF) determined across all samples by targeted deep sequencing at an average coverage of > 2500X. Three tumor regions had very low purity (< 0.05 based on the VAFs of somatic mutations) and were thus excluded from the data summary.

Across the 46 tumor samples with tumor purity > 0.05, the median mutation rate was 0.7/Mb (range 0.3–1.58/Mb; Additional file 3: Table S2), slightly higher than the 0.5/Mb (range 0.1–0.9/Mb) reported in previous studies [40]. Somatic copy number variations (CNVs), identified by SNP array or exome analysis, also exhibit intratumor heterogeneity (Additional file 2: Fig. S6). Somatic variants identified in each patient were classified as truncal (i.e. present in all sequenced tumor regions), shared (i.e. in ≥ 2 tumor regions but not all regions), or private (i.e. exclusive to a single tumor region). Truncal SNV/indel mutations had the lowest percentage across all patients (median of 18%, range 1.8–88%) (Fig. 1B) while truncal CNVs are more frequent (median of 44%, range 13–100%) (Fig. 1C). Interestingly, patients with longer treatment duration (> 100 days) have higher proportion of truncal CNVs compared with those with shorter treatment duration (median 0.85 versus 0.22, p value is 0.03 by Fisher’s exact test), indicating possible selection for specific CNV-defined lineages during treatment.

Considering previously reported driver genes discovered by genomic landscape mapping of DMG-H3 K27-a [21], all 11 patients had truncal histone mutations in *H3F3A* (n = 9) or *HIST1H3B *(*n* = 2), which co-occurred with mutations in *TP53* (n = 6 patients), *ACVR1* (n = 1), *PTPN11* (n = 2), or *PPM1D* (n = 2) (Fig. 1A). These co-occurring mutations were truncal except in case 325 which had two non-truncal *TP53* mutations (R248W, R273H) in different tumor regions. PI3K/MAPK pathway alterations occurred in every patient such as through activating mutations in *PIK3CA*, *PIK3R1*, and *PDGFRA;* loss of function mutations in *PTEN* and *NF1*; and focal amplification of *PDGFRA* and *MET*. All except for a focal amplification of *MET* in a single case were non-truncal variants, suggesting that activation of these pathways is a late event. Overall, the temporal order of mutational acquisition, inferred in our cohort was consistent with previous multi-region studies of DMG-H3 K27-a evolution [21–23, 41].

### High mutational prevalence and convergent evolution leading to activation of the PI3K pathway

Eight of 11 (72%) cases harbored at least one mutation in *PIK3CA* or *PIK3R1*, significantly higher (p value = 0.04 by Fisher’s exact test) than the 28–44% prevalence reported in previous studies [22, 23, 41]. Convergent evolution leading to activation of the PI3K pathway was found in four patients as different PI3K mutations were present in different tumor regions or co-existed in the same tumor region of the same patient (Fig. 1A; see asterisks and hash marks). The most striking pattern was found in case 756 where four distinct PI3K mutations were detected amongst the five tumor regions, including two SNVs in *PIK3CA* (H1047R and E545G) and two hotspot in-frame indels (D569ED and N453del) in *PIK3R1*. Interestingly, convergent evolution was observed only in patients with relatively short treatment duration (< 100 days) although the association was not significant due to small sample size (p value is 0.14 by Fisher’s exact test).

### Phylogeny and clonal diversity across multiple tumor regions

Tumor purity, estimated from the clonality of *H3F3A* or *HIST1H3B* K27M mutations, had a median of 0.8 across all 49 tumor regions (Additional file 3: Table S1) and was not significantly correlated with mutation burden (p value 0.15 by Pearson’s *r* test). Copy gain of histone H3.3 (*H3F3A*) via gain of chromosome 1 (chr1) or 1q, the chromosome arm where *H3F3A* is located, was present in 5 patients (i.e. cases 325, 311, 756, 309 and 313 in Additional file 2: Fig. S6), all duplicating the K27M mutant allele (see Additional file 2: Fig. S2 A-C for an example of modeling). 1q gain was also detected in the two cases (310 and 160) with a H3.1 (*HIST1H3B*) mutation.

**Fig. 2:**
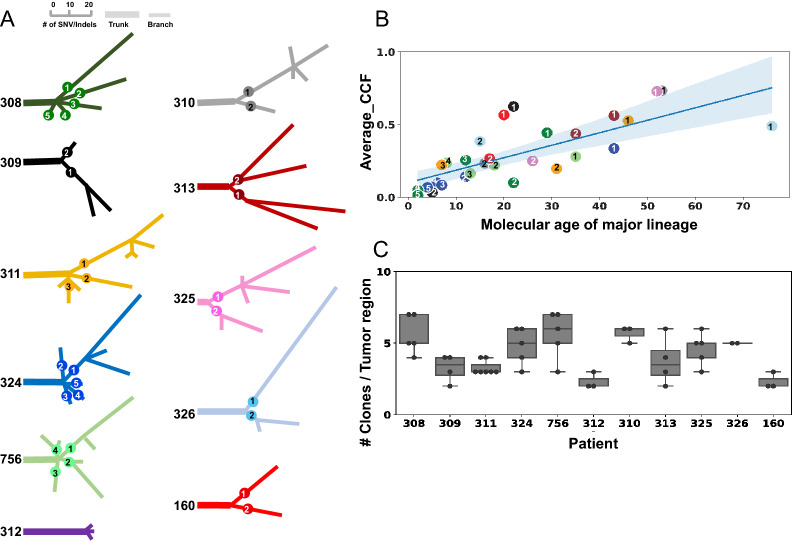
An overview of phylogeny and clonal diversity of the cohort. **A** An overview of phylogenetic trees constructed from somatic variants identified from multi-region tumor sequencing data. The lengths of branches are drawn proportional to the number of somatic SNVs/indels as indicated by the scale bar at the top. Each tree is labeled by patient ID and colored distinctively, and has its trunk drawn with a thick line while the branches are drawn with thinner lines. The immediate child branches of the trunk represent the main lineages which are labeled with a numeric number. **B** A significant correlation of the average CCF (y-axis) and the molecular age (x-axis) of major lineages identified in the 10 patients with multi-lineage phylogeny (R = 0.68, p value = 0.00016 by Pearson’s correlation test). Each dot represents a major lineage shown in panel A, labeled with the same numbering and color code. The average CCF was calculated by summing up the CCFs of all subclones descending from a major lineage divided by the number of tumor regions profiled in the patient. Regions with low tumor purity (< 5%) are excluded. The molecular age for each lineage is defined by the number of mutations from the trunk to the most distant descendant (node) for each lineage. **C** Distribution of number of subclones in tumor regions profiled in each patient shown as a boxplot

For each patient, a phylogenetic tree was constructed based on CCF of somatic mutations and CNVs across all tumor regions. An overview of phylogenetic trees for all patients is shown in Fig. 2A, where the lengths of branches are proportional to the number of point mutations representing their molecular age. Truncal variants (represented by thick lines in Fig. 2A) are present ubiquitously at all tumor regions, and form the genetic basis of a founder clone, a.k.a. the most recent common ancestor. These founders may spawn one or more child nodes (or branches) representing the major lineages (thin lines in Fig. 2A) of subclonal populations detected in each patient. Multiple lineages were found in all except one patient, case 312, who had the longest treatment duration and harbored a H3.1 mutation (Fig. 2A). Strikingly, there is a positive correlation between the average CCF and the molecular age (as defined by the number of mutations) of major lineages (R = 0.68, p value = 0.00016 by Pearson’s correlation test, Fig. 2B).

Amongst the 49 tumor regions, 25 were occupied by subclones descending from the same lineage while the remaining 24 were co-inhabited by those descending from admixed lineages. Amongst regions with multi-lineage occupancy, the predominant lineage accounted for 50–95% of CCF, indicating a high variability in the admixed subclonal populations (Additional file 3: Table S3). The median number of clones in each tumor region was 4 (range: 2–7, Fig. 2C). Considering all clones identified across multiple tumor regions for each patient, the median number of clones was 12 (range 4–15). The two patients with H3.1 mutations (160 and 312) had the lowest overall clonal diversity, with only 4 clones detected across the tumor regions. Interestingly, these two patients also lacked mutations in *TP53* and *PPM1D*. To further investigate this observation, we analyzed a larger cohort of published data [21] and found that the number of clones in a tumor sample was lower in H3.1-mutant cases with marginal significance (median 4.5 in H3.1 versus 6.0 in H3.3-mutant patients, p value = 0.056). This trend was not detected in samples with the *TP53/PPMID* mutation status.

### Clonal invasive patterns, extrapontine invasion and convergent evolution

**Fig. 3:**
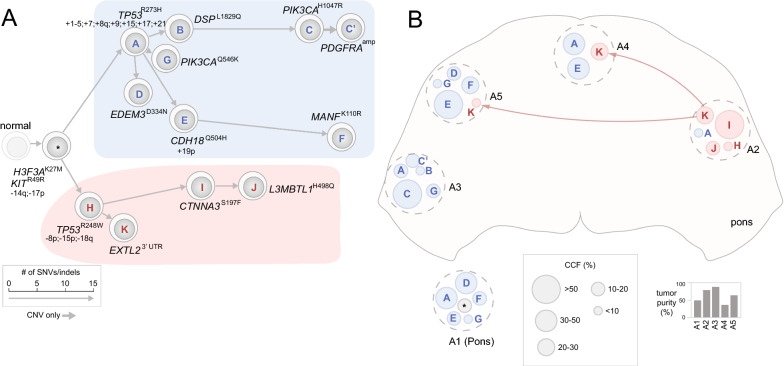
Early divergence defined by convergent evolution of TP53 mutations in patient 325. **A** A phylogenetic tree constructed from multi-region tumor samples with clones represented as cells and arrows indicating evolutionary branches. The founder clone comprised of truncal variants is shown in gray marked by a “*” from which the major branches are highlighted by distinct colors, i.e. the two major branches bearing TP53^R273H^ and TP53^R248W^ shown in blue and pink, respectively. The length of each branch is drawn proportional to the number of somatic SNVs/indels in its lineage-specific mutation cluster (details of the clusters are shown in Additional file 2: Fig. 7A). The clones are marked alphabetically by their corresponding mutation clusters with selected mutations labeled, along with chromosome/arm-level somatic CNV gains indicated with “+” and losses with “−”. **B** Clonal composition of each tumor region. Each tumor region is marked by a dotted gray circle placed to its spatial position viewed from the top (transverse plane). Clones within each region are shown as circles colored and labeled by their lineages on the phylogenetic tree shown in (**A**). A founder clone marked by “*” may also represent an unknown subclone with undetected private mutations. The diameter of each circle corresponds to the size of the clone (legend at bottom). A1 was a tumor region within pons with unknown position and is shown outside the pons. Invasion of clone K of pink branch from A2 is shown by pink arrows. Tumor purity of each sample is indicated as a bar graph below

**Fig. 4:**
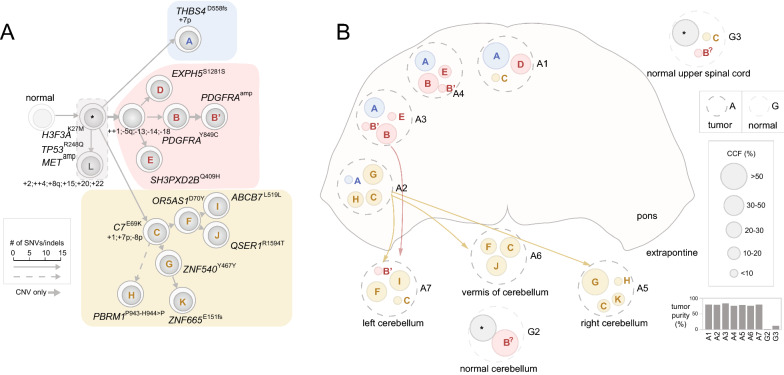
Extrapontine invasion to cerebellum and spinal cord involving tumor cells from different lineages in patient 311. The two panels are drawn using the same style as Fig. 3. **A** Truncal variants consist of a cluster representing the founder clone (*) and cluster L with varying CCF in different tumor regions which are outlined with a dotted line; the descending nodes can arise either from the founder clone (*) or clone A. The phylogenetic tree comprised three major branches including a branch (pink) initiated by 4 CNVs shown by a thick gray arrow. A dotted arrow indicates uncertain lineage of clone H which can be a descendant from either clone C or clone G. Bi-allelic duplication such as gain of chr4 on the trunk and gain of chr1 on the yellow branch are marked with + +. **B** Spatial position and clonal composition of seven tumor samples and two histologically normal samples (G2 and G3) that contained low-abundance tumor cells. Extrapontine invasions involving tumor cells from both the pink (clone B′) and yellow linages (clones C, G and H) are marked by pink and yellow arrows, respectively. The identity of clone B in normal samples is marked as B^?^ as it is impossible to determine the presence of a *PDGFRA* amplicon due to the low tumor content

**Fig. 5:**
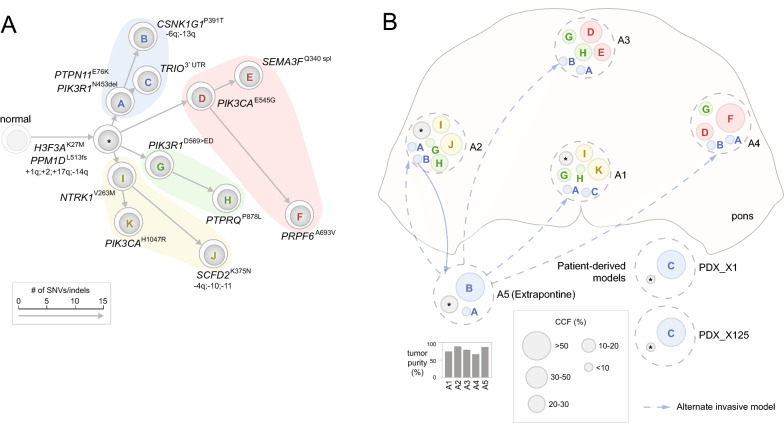
Extensive PI3K convergent evolution in patient 756. The two panels are drawn using the same style as Fig. 3. **A** Phylogenetic tree for patient 756 with four major branches each bearing a distinct PI3K mutation. **B** Spatial position and clonal composition of 5 profiled tumor regions including an extrapontine sample of unknown location (A5). Extrapontine invasions involving tumor cells from blue (clone A, B) are marked by blue arrows. Alternatively, the dotted blue arrows indicate that presence of clones A nd B in tumor regions within the pons could be caused by invasion from the extrapontine region A5. Two PDX samples (PDX_X1 and PDX_X125) derived from A1 shown a monoclonal lineage as only clone C was detected in both models

By superimposing the clonal compositions onto the anatomical locations of each tumor region, we were able to depict the spatial occupancy pattern in each case, unveiling invasion of tumor cells within the pons and into extrapontine regions. Prominent patterns of invasion projected from admixed subclonal population in three representative cases are presented below (Figs. 3, 4, 5), with tumor samples acquired at autopsy from different regions of the same patient labeled arbitrarily as A1, A2, etc., and the histologically normal samples labeled as G1, G2, etc. Four extrapontine normal samples from two cases (311 and 326) bore low-level H3.3 (*H3F3A*) K27M mutations, which were further assessed for their tumor clonal composition (Additional file 1: Supplementary Methods, Additional file 2: Fig. S5).

#### Patient 325: early divergence defined by convergent evolution of TP53 mutations

Patient 325 was under crenolanib treatment for 27 days and was the only patient with two distinct somatic *TP53* mutations, R273H and R248W, which were identified in the five autopsy samples analyzed. The presence of these different mutations was distinct spatially and associated with highly divergent CNV patterns (Additional file 2: Fig. S7A, B). Specifically, tumor regions A1 and A3 had clonal TP53^R273H^ accompanied by extensive copy number gain affecting > 50% of the cancer genomes. By contrast, the other three regions had an admixture of TP53^R273H^ and TP53^R248W^ mutations; one of these regions (A2) had a dominant clone of TP53^R248W^ (CCF > 0.80) accompanied by absence of aneuploidy.

The phylogenetic tree of this patient had a very short trunk comprised of only two founder mutations in *H3F3A* and *KIT* as well as loss of 14q and 17p (Fig. 3). While 14q loss was omnipresent with an estimated clonality matching the tumor purity in each region (Additional file 2: Fig. S2D, E), CNVs on chromosome 17 had varying patterns of gain and loss at different tumor regions. Therefore, the timing of 17p loss was inferred based on fitting the empirical data against models for early versus late acquisition of this event (Additional file 1: Supplementary Methods, Additional file 2: Fig. S3). The tree had two divergent branches bearing TP53^R273H^ and TP53^R248W^ (shaded blue and pink, respectively in Fig. 3A), representing two lineages resulting from an early divergence in tumor evolution. The TP53^R273H^ lineage (labeled as lineage 1 in Fig. 2A) was twice as long as that of TP53^R248W^ (labeled as lineage 2 in Fig. 2A) indicating an older molecular age. This inferred molecular age is consistent with the overall dominance of the TP53^R273H^ lineage (marked in blue in Fig. 3B) as its average CCF is thrice as high as that of TP53^R248W^ lineage (0.75 versus 0.25) in this patient.

Tumor regions A1 (location unknown) and A3 were occupied exclusively by the TP53^R273H^ lineage. The predominant clone in A3 (clone C in Fig. 3, CCF 0.7) had acquired additional PI3K/MAPK pathway mutations (PIK3CA^H1047R^ and a focal *PDGFRA* amplification, Additional file 2: Fig. S7C) evolutionarily late. The *PDGFRA* amplicon was estimated to have a CCF of ~ 0.20 with ~ 5-copy gain based on modeling of VAF of the co-amplified KIT truncal mutation. The other three regions, A2, A4, and A5, were co-occupied by tumor cells from both TP53^R273H^ and TP53^R248W^ lineages with varying CCFs (Fig. 3B). A2, located opposite A3, was the only region dominated by the TP53^R248W^ lineage; it also had ~ 5% of admixture of tumor cells from the TP53^R273H^ branch. A4 and A5 were predominantly occupied by descendants of the TP53^R273H^ branch but had 25% and ~ 5% of TP53^R248W^ admixture, respectively; and the level of admixture was correlated with regional proximity to A2. The admixed subclonal populations in these tumor regions suggest two waves of occupation—one by the TP53^R273H^ branch and another by the TP53^R248W^ branch likely initiated at or near A2. The molecular age, inferred from the lineage-specific mutation count, indicates that TP53^R273H^ lineage occupation within the pons may have preceded the TP53^R248W^ lineage.

#### Patient 311: extrapontine invasion to cerebellum and spinal cord involving tumor cells from different lineages

Patient 311 was treated for 192 days and had the highest number of samples profiled which included 7 tumors (A1-A7) and 3 normal samples (G1-G3). The anatomical sites of tumor samples included the pons (A1-A4) and cerebellum (A5-A7 representing right cerebellum, vermis and left cerebellum, respectively) while the normal samples were collected from the frontal cortex, cerebellum and upper spinal cord. Extensive aneuploidy was present in all tumor samples, some of which was caused by a bi-allelic duplication resulting an admixture of 2-copy and 4-copy chromosomes, including a gain of chromosome 4 (Additional file 2: Fig. S8).

The phylogenetic tree had a trunk with a substantive number of somatic alterations including 15 point mutations (e.g. H3F3A^K27M^, TP53^R248Q^, and MET^P657T^), *MET* amplification, 17p LOH and copy number gains of 6 chromosomes or chromosome arms (Fig. 4A, Additional file 2: Fig. S8B). Eight truncal variants were subclonal in all tumor regions (0.5 ≤ CCF < 1.0, Additional file 1: Supplementary Methods), none of which were in known DMG-H3 K27-a driver genes (cluster L in Fig. 4A, Additional file 2: Fig. S8B). The tree had three major branches, two of which were long branches separated from the trunk by > 30 point mutations (colored blue and yellow in Fig. 4) while the third was a short branch with < 10 point mutations (colored pink in Fig. 4). Lineage-specific CNVs were detected in all three major branches; notable events included independent 7p gain in the two long branches with contrasting allelic specificity (Additional file 2: Fig. S8B, inset 1) and gross copy-loss of 4 chromosomes (full or at arm’s level) seeding the short branch.

Within the short branch, one sub-branch was established by a mutation cluster, denoted cluster B, which contained a PDGFRA^Y849C^ mutation altering the known phosphorylated tyrosine residue Y849 in PDGFRA[42] (Additional file 2: Fig. S8A). By an integrated modeling (Additional file 1: Supplementary Methods, Additional file 2: Fig. S4), we found that the focal amplification, an event occurring later than PDGFRA^Y849C^, is in fact an amplicon of PDGFRA^Y849C^ which increased the dosage of the mutant allele by sevenfold (Additional File 2: Fig. S8D). A node B’ was added to this branch to mark this late amplification event.

Tumor cells of the two long branches showed different patterns of spatial occupancy. The one bearing C7^E69K^ (colored yellow in Fig. 4) had extensive sub-branches and was found to dominate all three extrapontine regions (i.e. A5-7 of left/right cerebellum and vermis), but only 1 out of the 4 regions within the pons (i.e. A2). This pattern indicates that the branch likely initiated at A2 within the pons followed by an invasion leading to clonal occupancy and continued evolution in the three extrapontine regions (Fig. 4B). By contrast, the THBS4^D558fs^ bearing long branch (colored blue in Fig, 4) was present exclusively within the pons but admixed with tumor cells from the other branches. The short branch (colored pink in Fig. 4) was primarily present within the pons with tumor cells from the PDGFRA^Y849C^ sub-branch (denoted B) accounted for a CCF of ~ 0.4 in A3 and A4. Interestingly, a subclone comprised entirely of tumor cells containing the PDGFRA^Y849C^ amplicon (marked B’ in Fig. 4) was admixed with subclones of C7^E69K^ branch at the extrapontine sample A7 (left cerebellum) indicating that an extrapontine invasion occurred after clone B acquired the PDGFRA^Y849C^ amplicon. Therefore, two waves of extrapontine invasion likely occurred—one by the long C7^E69K^ branch and another by a descendant of the short branch carrying the PDGFRA^Y849C^ amplicon.

Two of the three normal samples, G2 (normal cerebellum) and G3 (normal upper spinal cord), also harbored truncal mutations and were estimated to contain 2% and 12% tumor cells, respectively, based on the VAF of *H3F3A* K27M mutation from deep sequencing data (Additional file 2: Fig. S5A, Additional file 1: Supplementary Methods). PDGFRA^Y849C^ cells (mutation cluster B in Fig. 4) were detected at G2 and G3 with a high (0.50) and a low (0.10) CCF, respectively. G3 also contained admixed tumor cells from the C7^E69K^ branch (mutation cluster C in Fig. 4) at the CCF of 0.10 (Fig. 4B, Additional file 2: Fig. S5B, C). Therefore, these “contaminating” tumor-in-normal cells corroborated the extrapontine invasion patterns detected in the extrapontine tumor regions.

#### Patient 756: extensive PI3K convergent evolution in primary tumors but monoclonal in PDX models

Patient 756 was treated for 24 days, and the phylogenetic tree of this patient was constructed from five tumor samples, four within the pons (A1–A4) and one extrapontine of unknown location (A5). In addition, we incorporated mutations from two PDX samples derived from A1 to verify and refine the clonal lineage and composition from the patient samples. The tree had a trunk comprised of 11-point mutations (e.g. H3F3A^K27M^, and PPM1D^L523fs^) as well as copy gains of 1q, 2 and 17q, and copy loss of 14q (Fig. 5A, Additional file 2: Fig. S9). The most striking feature of the tree is the presence of a distinct mutation in the PI3K pathway in each of its four major branches, thereby representing the most diverse example of PI3K convergent evolution in our cohort (Fig. 5A). Three of the four branches originated with a distinct PI3K pathway mutation (i.e. PIK3R1^N453del^, PIK3CA^E545G^ and PIK3R1^D569>ED^ for the branches colored blue, pink and green, respectively, in Fig. 5) while the fourth (yellow in Fig. 5) had one sub-branch with PIK3CA^H1047R^.

While the four pontine tumors had an admixture of 3 out of the 4 branches, the extrapontine tumor A5 was occupied by tumor cells from a single lineage, i.e. those descending from the shortest (blue in Fig. 5) branch with a CCF of 0.80 along with a minor population of the founder clone. The shortest branch had two lineage-specific mutations PIK3R1^N453del^ and PTPN11^E76K^ that are known to activate PI3K and RAS signaling pathways, respectively (Fig. 5). Notably, descendants from this branch were also present in all four pontine tumors as minor subclones (combined CCF ranging 0.10–0.15). The exclusive extrapontine-dominance of this branch implies a potential extrapontine origin, i.e. after an early escape of the ancestral clone from the pons. However, accounting for its broad prevalence within the pons implies a broad “reciprocal” invasion back into the pons, a phenomenon not supported by published literature. Alternatively, the extrapontine dominance can be explained by escape of a subclone originated within the pons, but having high migratory/invasive capabilities and an extraordinary fitness in the extrapontine region. Interestingly, the fitness advantage of descendants from this branch can also be discerned through the mutation profile of the two PDX models established from A1: a minor subclone with an estimated CCF of < 0.10 at A1 from this branch is the predominant clone accounting for > 0.90 CCF in both models (clone C in Fig. 5).

## Other cases: extrapontine invasion, single-lineage and PI3K evolution patterns

### Extrapontine invasion to medulla and midbrain detected in normal brain samples

Case 326 is another patient whose normal samples (G3 and G4 derived from medulla and midbrain, respectively) contained a low fraction (~ 10%) of the tumor cells (Additional file 2: Fig. S10). The lineage of the tumor cells in these normal samples could be tracked to the trunk and the short branch (Additional file 2: Fig. S10), indicating multiple waves of extrapontine invasion, to medulla (G3) and midbrain (G4).

Predominance of a single lineage across all tumor regions was identified in patients 309 (Additional file 2: Fig. S11) and 310 (Additional file 2: Fig. S12) while only a single lineage was detected in patient 312 who had the longest treatment duration (> 600 days) in our cohort (Additional file 2: Fig. S13). The predominant lineage in 309 harbored PIK3CA^H1047R^ mutation while that of case 310 had a sub-branch with a subclonal *PDGFRA* amplification present in all tumor regions.

PI3K mutations were detected in four additional patients including single-lineage evolution in patients 308 (Additional file 2: Fig. S14) and 324 (Additional file 2: Fig. S15), and convergent evolution in patients 160 and 313. Patient 160, despite having the lowest clonal diversity in the entire cohort (i.e. 3 clones identified amongst the three tumor samples profiled), had two independent *PIK3CA* mutations, including PIK3CA^Q546K^ in A1 and PIK3CA^E545K^ in A2 and A3 (Additional file 2: Fig. S16). One of the major branches of case 313 had two sub-branches with distinct mutations in *PIK3CA* and *PIK3R1* (Additional file 2: Fig. S17).

## Discussion

Our multi-region tumor profiling of DMG-H3 K27-a autopsy samples from 11 patients treated with crenolanib revealed a temporal order of mutational acquisition similar to previously published multi-region studies [21–23, 41]. Several findings unique in our cohort unveil new insights on DMG-H3 K27-a intratumor heterogeneity. First, the > 70% prevalence of PI3K pathway mutations in our studies is significantly higher than prior studies. This suggests PI3K activation may be more prevalent in DMG-H3 K27-a than previously recognized. Second, convergent evolution of PI3K pathway mutations was detected in 4 patients (~ 40%), all of whom had short treatment duration (24–79 days). By contrast, 3 out of the 4 single-lineage mutations were found in patients with long treatment duration (174–191 days). This indicates a possibility for selecting a specific PI3K mutation under crenolanib treatment. However, significance of this pattern cannot be assessed due to the small size of the patient cohort. Third, the higher proportion of truncal CNVs in patients with long treatment history indicates selection for a specific lineage during treatment. Unfortunately, this could not be evaluated rigorously as all tumor samples profiled were obtained at autopsy of treated patients. Consequently, we were not able to identify mutational profile and invasion patterns associated with crenolanib treatment due to this limitation. Future studies designed to profile both untreated and post-treatment tissues would help to illuminate whether these mutations were pre-existing or acquired during treatment.

Nearly half of the cases showed extensive aneuploidy with copy losses and gains affecting > 50% of the tumor genomes, making it imperative to incorporate CNV clonality into our analysis of tumor purity and CCF of the mutational clusters (Additional file 2: Fig. S6). The resulting data also enabled tracking the lineage of subclonal CNVs, which we believe is an important component of DMG-H3 K27-a intratumor heterogeneity, as extensive CNV heterogeneity was previously unveiled by single-cell RNA-seq of two DMG-H3 K27-a tumors [14]. We did not exclude any mutation clusters, as those of small size can be critical in constructing the phylogeny. For example, the founder clone in patient 325 is comprised of two point mutations (Fig. 3) while a minor subclone distinguishable from its parent by only 1 SNV becomes the predominant population in the two PDX models established from case 756 (Fig. 5).

We noted that the purity estimates incorporating the founder mutation H3 K27M differed from those analyzed by popular tools such as ASCAT [34] and ABSOLUTE [24] (Addition file 3: Table S4). This may reflect that the assumption of a uniformed ploidy implemented by these programs may not be robust when modeling admixtures of tumor cells with different ploidy profile (e.g. sample 325_A4 in Fig. 3). Our model-based approach not only helps to resolve ambiguity in variants with uncertain lineage (e.g. the 17p loss in case 325, Addition file 2: Fig. S7), it can also lead to interesting findings. For example, we identified copy gains of the *H3F3A* mutant allele, rather than the wild-type allele, in all five patients with both *H3F3A* mutation and chr1 or 1q gain, indicating that increasing the dosage of the *H3F3A* mutant allele may affect tumorigenesis or progression. Interestingly, 1q gain may have an impact beyond the *H3F3A* mutant allele, as it was also found in two patients who had *HIST1H3B* mutation (located on chromosome 6) instead of *H3F3A* mutation via single-copy gain (case 160) or bi-allelic duplication (case 312). Our modeling of *PDGFRA* amplification present in four cases showed that these are subclonal events with high-level amplification reminiscent of those caused by extrachromosomal DNA (ecDNA) or homogeneous staining region (HSR). In patient 311, our modeling showed that *PDGFRA* amplification was a de facto PDGRFA^Y849C^ amplicon (Additional file 2: Fig. S8)—similar amplification-associated resistance mechanism was previously unveiled by in vitro cell-line or in vivo PDX model based experiments [43], suggesting this event may be relevant to therapeutic resistance.

Extrapontine tumor samples were profiled for two patients (311 and 756), and their clonal lineages can be tracked to at least one tumor region within the pons. Therefore, extrapontine invasion, previously considered an early event [21], can also involve late-evolving subclones. This is best illustrated by the presence of a subclone with the PDGFRA^Y849C^ amplicon, projected to be a late event based on our modeling, in the cerebellum of patient 311 (Additional file 2: Fig. S8). Interestingly, two of the four normal brain samples from this patient contained tumor-in-normal contamination, and the subclonal identity of the “contaminants” matches those detected in the extrapontine tumor samples including the one containing PDGFRA^Y849C^. Therefore, the “contaminating tumor cells” in normal samples are analogous to trails of footprints left by extrapontine migration. Indeed, in all four such normal tissues not only founder clones but also their descendant subclones were detected, an observation consistent with a multi-wave invasion hypothesis (Fig. 4 and Additional file 2: Figs. S8 and S10). Overall, co-occupancy of tumor cells from different lineages was detected in ~ 50% (24/49) of the tumor regions profiled, indicating DMG-H3 K27-a invasion can be an ongoing process driven by continued clonal evolution and expansion.

Our study unveils important insights into DMG-H3 K27-a evolution and invasion. The temporal order of tumor clone invasion was inferred from modeling of mutational acquisition (e.g. PDGFRA^Y849C^ amplicon) as well as the relative molecular age of the major branches of the founder clone. The significant correlation between the average CCF and the molecular age in our cohort (Fig. 2A) provides support for this temporal inference, as a more expansive occupancy is likely driven by a lineage with longer evolution time. Due to lack of information on the precise location of the autopsy samples, we were unable to incorporate additional longitudinal data such as clinical imaging into our analysis, which could provide a definitive answer regarding invasions driven by multiple timepoints versus collaborative co-migration of clones from different lineages. Future studies designed with a goal of integrating longitudinal imaging data with genomics-based tumor clonal evolution analysis can potentially provide a more refined view of DMG-H3 K27-a evolution in response to therapeutic treatment.

## Supplementary Information

**Additional file1.** Supplementary methods.

**Additional file2.** Supplementary figures.

**Additional file3.** Supplementary tables.

## Data Availability

All data generated or analyzed in this manuscript are included in the article and its supplementary material. All request for data or supporting material may be sent to the corresponding author. Exome sequencing BAM files are available on St. Jude Cloud as part of the Pediatric Cancer Genome Project and can be accessed under the accession SJC-DS-1018 (https://platform.stjude.cloud/data/cohorts?dataset_accession=SJC-DS-1018).

## Abbreviations

DMG-H3 K27-a: H3 K27-altered diffuse midline gliomas
PDGFRA: Platelet-derived growth factor receptor alpha
CNV: Copy number variations
WES: Whole exome sequencing
SNV: Single nucleotide variants
Indels: Small insertions/deletions
VAF: Variant allele fraction
BAF: B-allele fractions
SNPs: Single nucleotide polymorphisms
LOH: Loss-of-heterozygosity
CCF: Cancer cell fractions
PI3K: Phosphatidylinositol 3-kinases
AI: Allelic Imbalance

## References

[1] Louis DN, Perry A, Wesseling P, Brat DJ, Cree IA, Figarella-Branger D (2021). The 2021 WHO classification of tumors of the central nervous system: a summary. Neuro Oncol.

[2] Hoffman LM, Van Zanten SEMV, Colditz N, Baugh J, Chaney B, Hoffmann M (2018). Clinical, radiologic, pathologic, and molecular characteristics of long-term survivors of diffuse intrinsic pontine glioma (DIPG): a collaborative report from the International and European Society for Pediatric Oncology DIPG registries. J Clin Oncol.

[3] Hargrave D, Bartels U, Bouffet E (2006). Diffuse brainstem glioma in children: critical review of clinical trials. Lancet Oncol.

[4] Ramos A, Hilario A, Lagares A, Salvador E, Perez-Nuñez A, Sepulveda J (2013) Brainstem Gliomas. Semin Ultrasound, CT MRI 10.1053/j.sult.2013.01.001 23522775

[5] Robison NJ, Kieran MW (2014). Diffuse intrinsic pontine glioma: a reassessment. J Neurooncol.

[6] Warren KE (2012). Diffuse intrinsic pontine glioma: poised for progress. Front Oncol.

[7] Schwartzentruber J, Korshunov A, Liu XY, Jones DTW, Pfaff E, Jacob K (2012). Driver mutations in histone H3.3 and chromatin remodelling genes in paediatric glioblastoma. Nature.

[8] Buczkowicz P, Hoeman C, Rakopoulos P, Pajovic S, Letourneau L, Dzamba M (2014). Genomic analysis of diffuse intrinsic pontine gliomas identifies three molecular subgroups and recurrent activating ACVR1 mutations. Nat Genet.

[9] Wu G, Broniscer A, McEachron TA, Lu C, Paugh BS, Becksfort J (2012). Somatic histone H3 alterations in pediatric diffuse intrinsic pontine gliomas and non-brainstem glioblastomas. Nat Genet.

[10] Wu G, Diaz AK, Paugh BS, Rankin SL, Ju B, Li Y (2014). The genomic landscape of diffuse intrinsic pontine glioma and pediatric non-brainstem high-grade glioma. Nat Genet.

[11] Fontebasso AM, Papillon-Cavanagh S, Schwartzentruber J, Nikbakht H, Gerges N, Fiset PO (2014). Recurrent somatic mutations in ACVR1 in pediatric midline high-grade astrocytoma. Nat Genet.

[12] Taylor KR, Mackay A, Truffaux N, Butterfield YS, Morozova O, Philippe C (2014). Recurrent activating ACVR1 mutations in diffuse intrinsic pontine glioma. Nat Genet.

[13] Dai C, Celestino JC, Okada Y, Louis DN, Fuller GN HE. PDGF autocrine stimulation dedifferentiates cultured astrocytes and induces oligodendrogliomas and oligoastrocytomas from neural progenitors and astrocytes in vivo 10.1101/gad.903001 PMC312748 11485986

[14] Filbin MG, Tirosh I, Hovestadt V, Shaw ML, Escalante LE, Mathewson ND (2018). Developmental and oncogenic programs in H3K27M gliomas dissected by single-cell RNA-seq. Science.

[15] Larson JD, Kasper LH, Paugh BS, Jin H, Wu G, Kwon CH (2019). Histone H33 K27M accelerates spontaneous brainstem glioma and drives restricted changes in bivalent gene expression. Cancer Cell.

[16] Paugh BS, Zhu X, Qu C, Endersby R, Diaz AK, Zhang J (2013). Novel oncogenic PDGFRA mutations in pediatric high-grade gliomas. Cancer Res.

[17] Tinkle CL, Broniscer A, Chiang J, Campagne O, Huang J, Orr BA (2021). Phase 1 study using crenolanib to target PDGFR kinase in children and young adults with newly diagnosed DIPG or recurrent high-grade glioma, including DIPG. Neuro-Oncol Adv.

[18] Rusch M, Nakitandwe J, Shurtleff S, Newman S, Zhang Z, Edmonson MN (2018). Clinical cancer genomic profiling by three-platform sequencing of whole genome, whole exome and transcriptome. Nat Commun.

[19] Ma X, Shao Y, Tian L, Flasch DA, Mulder HL, Edmonson MN (2019). Analysis of error profiles in deep next-generation sequencing data. Genome Biol.

[20] Gerlinger M, Rowan AJ, Horswell S, Larkin J, Endesfelder D, Gronroos E (2012). Intratumor heterogeneity and branched evolution revealed by multiregion sequencing. N Engl J Med.

[21] Vinci M, Burford A, Molinari V, Kessler K, Popov S, Clarke M (2018). Functional diversity and cooperativity between subclonal populations of pediatric glioblastoma and diffuse intrinsic pontine glioma cells. Nat Med.

[22] Hoffman LM, DeWire M, Ryall S, Buczkowicz P, Leach J, Miles L (2016). Spatial genomic heterogeneity in diffuse intrinsic pontine and midline high-grade glioma: implications for diagnostic biopsy and targeted therapeutics. Acta Neuropathol Commun.

[23] Nikbakht H, Panditharatna E, Mikael LG, Li R, Gayden T, Osmond M (2016). Spatial and temporal homogeneity of driver mutations in diffuse intrinsic pontine glioma. Nat Commun.

[24] Carter SL, Cibulskis K, Helman E, McKenna A, Shen H, Zack T (2012). Absolute quantification of somatic DNA alterations in human cancer. Nat Biotechnol.

[25] Jamal-Hanjani M, Wilson GA, McGranahan N, Birkbak NJ, Watkins TBK, Veeriah S (2017). Tracking the evolution of non-small-cell lung cancer. N Engl J Med United States.

[26] Ma X, Edmonson M, Yergeau D, Muzny DM, Hampton OA, Rusch M (2015). Rise and fall of subclones from diagnosis to relapse in pediatric B-acute lymphoblastic leukaemia. Nat Commun.

[27] Li H (2010) Aligning new-sequencing reads by BWA BWA: Burrows-Wheeler Aligner. Slides

[28] Edmonson MN, Zhang J, Yan C, Finney RP, Meerzaman DM, Buetow KH (2011). Bambino: a variant detector and alignment viewer for next-generation sequencing data in the SAM/BAM format. Bioinformatics.

[29] Zhang J, Ding L, Holmfeldt L, Wu G, Heatley SL, Payne-Turner D (2012). The genetic basis of early T-cell precursor acute lymphoblastic leukaemia. Nature.

[30] Hagiwara K, Edmonson MN, Wheeler DA, Zhang J (2021). indelPost: harmonizing ambiguities in simple and complex indel alignments. Bioinformatics.

[31] McCarroll SA, Kuruvilla FG, Korn JM, Cawley S, Nemesh J, Wysoker A (2008). Integrated detection and population-genetic analysis of SNPs and copy number variation. Nat Genet.

[32] Talevich E, Shain AH, Botton T, Bastian BC (2016). CNVkit: genome-wide copy number detection and visualization from targeted DNA sequencing. PLOS Comput Biol.

[33] Tarabichi M, Salcedo A, Deshwar AG, Ni Leathlobhair M, Wintersinger J, Wedge DC (2021). A practical guide to cancer subclonal reconstruction from DNA sequencing. Nat Methods.

[34] Van Loo P, Nordgard SH, Lingjærde OC, Russnes HG, Rye IH, Sun W (2010). Allele-specific copy number analysis of tumors. Proc Natl Acad Sci.

[35] Roth A, Khattra J, Yap D, Wan A, Laks E, Biele J (2014). PyClone: statistical inference of clonal population structure in cancer. Nat Methods.

[36] Jiao W, Vembu S, Deshwar AG, Stein L, Morris Q (2014). Inferring clonal evolution of tumors from single nucleotide somatic mutations. BMC Bioinform.

[37] Jakubek YA, San Lucas FA, Scheet P (2019). Directional allelic imbalance profiling and visualization from multi-sample data with RECUR. Bioinformatics.

[38] Team RC (2013). R: A language and environment for statistical computing.

[39] McKinney W (2010) Data structures for statistical computing in python. In: Proc 9th Python Sci Conf. Austin, TX. p. 51–6

[40] Gröbner SN, Worst BC, Weischenfeldt J, Buchhalter I, Kleinheinz K, Rudneva VA (2018). The landscape of genomic alterations across childhood cancers. Nat England.

[41] Koschmann C, Farooqui Z, Kasaian K, Cao X, Zamler D, Stallard S (2017). Multi-focal sequencing of a diffuse intrinsic pontine glioma establishes PTEN loss as an early event. npj Precis Oncol.

[42] Heldin C-H, Lennartsson J (2013). Structural and functional properties of platelet-derived growth factor and stem cell factor receptors. Cold Spring Harb Perspect Biol.

[43] Ercan D, Zejnullahu K, Yonesaka K, Xiao Y, Capelletti M, Rogers A (2010). Amplification of EGFR T790M causes resistance to an irreversible EGFR inhibitor. Oncogene.

