# Supplementary material for: Convergent evolution and multi-wave clonal invasion in H3 K27-altered diffuse midline gliomas treated with a PDGFR inhibitor
Source: Acta Neuropathol Commun. 2022 May 31;10:80. doi: 10.1186/s40478-022-01381-0 (PMC9153212; doi:10.1186/s40478-022-01381-0)
Supplement: Supplementary file 1 — Additional file1. Supplementary methods. [file 40478_2022_1381_MOESM1_ESM.docx]

**Additional Methods for**

**Convergent evolution and multi-wave clonal invasion in H3 K27-altered diffuse midline gliomas treated with a PDGFR inhibitor**

Sasi Arunachalam*^1^, Karol Szlachta*^1^, Samuel W. Brady^1^, Xiaotu Ma^1^, Bensheng Ju^1^, Bridget Shaner^1^, Heather L. Mulder^1^, John Easton^1^, Benjamin J. Raphael^2^, Matthew Myers^2^, Christopher Tinkle^3^, Sariah J Allen^4^, Brent A. Orr^5^, Cynthia J. Wetmore^6#^, Suzanne J. Baker^7#^ and Jinghui Zhang^1#^

Departments of Computational Biology^1^, Radiation Oncology^3^, Pathology^5^, Developmental Neurobiology^7^, St. Jude Children's Research Hospital, Memphis, Tennessee, USA

^2^Department of Computer Science, Princeton University, Princeton, NJ 08540

^4^Thermofisher

^6^Neoleukin, Seattle, WA, USA

The authors declare no potential conflicts of interest.

*These authors contributed equally

#Correspondence to: [cwetmore@neoleukin.com](mailto:cwetmore@neoleukin.com); [Suzanne.Baker@StJude.org](mailto:Suzanne.Baker@StJude.org); [Jinghui.Zhang@StJude.org](mailto:Jinghui.Zhang@StJude.org)

This Addition file contains:

1. Supplementary Methods
2. Supplementary Table legends
3. **Supplementary Methods**

***Calibrate cellular prevalence (CP) of somatic copy number variations***

With the exception of bi-allelic duplication, somatic CNV or copy-neutral loss-of-heterozygosity (LOH) causes a deviation of B-allele fractions (BAFs) of germline heterozygous single nucleotide polymorphisms (SNPs) from the expected BAF of 0.5 in autosomes. The extent of the deviation, refer to as allelic imbalance (AI), is dependent on the cellular prevalence (CP, representing the fraction of all cells—whether tumor or normal—in the sample with the CNV) of the CNV. We used germline SNPs with BAFs of 0.4-0.6 identified from normal WES (using Bambino [1]), along with copy number data, to adjust the copy number value so that diploid regions (the lowest copy state lacking AI) would have an absolute copy value of ~2.0 and to determine the CPs of somatic CNVs.

Using 1-copy gain as an example, the BAF for a germline heterozygous SNP with the B-allele on the amplified and unamplified haplotype is expected to be respectively:

$$b_{1}=\frac{1+k}{2+k}, b_{2}=\frac{1}{2+k}$$

where *k* represents the CP of the CNV. The analytical process for 1-copy gain, 1-copy loss and copy-neutral (cn) LOH is very similar, following same steps.. The expected values allow construction of a BAF (*b*) distribution of all germline heterozgyous SNPs within a CNV segment in a tumor as follows:

$$\varphi_{k,s}\left( b \right)=a*G\left( b, b_{1}\left( k \right),s \right)+(1-a)*G\left( b, b_{2}(k),s \right)$$

Here, G represents the Gaussian distribution; *s* is the width of the distribution, which is largely dependent on coverage; and *a* is the proportion of SNPs with an amplified B-allele. The CP *k* can be obtained by fitting the cumulative distribution, defined above, to the empirical data, i.e. BAFs of germline SNPs in tumor exome or deep sequencing data (using scipy.optimize.curve_fit in python) so that the adjusted copy number *cn = 2 + k* is consistent with the observed BAF distribution in the tumor.

To estimate the accuracy of this approach, we simulated CNV CP in the range of 5-95% for two chromosomes (chr12 and chr17) known to have clonal 1-copy gain in the cancer cell line COLO-829 by sampling exome data generated from this cancer cell line and its matched normal COLO-829BL[2]. Reads from tumor and normal extracted at a proportion corresponding to CPs of 10% to 90% with 10% increment plus 5% and 95% were next used to estimate the CP of 1-copy gains by the above-mentioned BAF-based method. Each tumor fraction was sampled ten times to ensure robustness of the results. The BAF-based approach has a high precision (within 2%) in assessing the CP of CNVs that is present in at least 10% (i.e. k ≥ 0.1) of the cells in the sample (data not shown).

We used this approach to manually readjust the copy profiles identified by SNP array and an example of BAF-adjusted copy plots from tumor 325_A5 can be found in Additional File 2: Figure S1 which shows the global view of the original copy profile from SNP array, the BAF-adjusted copy profile, and the BAF distribution of germline heterozygous SNPs from 3 representative chromosomes with 1-copy loss, 1-copy gain and normal ploidy in the tumor.

***Estimate tumor purity using H3 K27M found mutation***

As copy-number gains at the *H3F3A* or *HIST1H3B* loci were present in ~50% of the tumors, we modeled histone H3 K27M variant allele fraction (VAF) against different CNV models and selected the model that matches the empirical data. An example is shown in Additional File 2: Figure S2, demonstrating how tumor purity was determined for the four tumor regions profiled by SNP array in case 325.

Case 325 has the *H3F3A* K27M founder mutation which is accompanied by a 1-copy gain in 3 out of the 4 tumor regions. As the K27M mutation is expected to be present in every tumor cell, a simplified version of the general model described in Material and Methods section was developed for evaluating three possible scenarios (Additional File 2: Figure S2A): A) a 1-copy gain amplifies the K27M mutation; B) a 1-copy gain amplifies the wild-type allele; and C) a 2-copy gain caused by a bi-allelic duplication followed by loss of the wild-type allele. Tumor purity (*t*) and the CCF of tumor cells with 2 copies vs 3 copies under each model is depicted in Additional File 2: Figure S2B which can affect both CNV (*c*) and VAF (*v*) of the K27M mutation. Using the BAF-adjusted CNV and VAF of K27M mutation, we can deduce the *t* (purity) and *f* (fraction of triploid tumor cells) under each model with the assumption that a correct model is expected to have both purity *t* and *f* ≤ 1.0. As shown in Additional File 2: Figure S2C, this condition was satisfied across all tumor samples only by model A (Additional File 2: Figure S2C) projecting their tumor purity ranging from 0.26 (325_A4) to 0.78 (325_A2 and 325_A3).

To verify the purity estimate based on H3F3A K27M mutation, we checked the 1-copy loss of chr14, another truncal variant present in all tumor regions of case 325. The CP of this event, *k*, can be estimated using the BAF-based method described in the section of “Copy number analysis”. This resulting value, ranging from 0.16 (325_A4) to 0.84 (325_A3), is consistent with *t* estimated from the K27M mutation under model A (Additional File 2: Figure S2D).

We also compared tumor purity estimate by this approach with ABSOLUTE[3], a popular algorithm that uses somatic CNVs for inferring tumor purity. In sample 325_A4, which had the lowest VAF (0.184) for *H3F3A* K27M among the four tumor regions in the patient, ABSOLUTE estimated the highest purity of 0.66 and predicted the tumor to be diploid. By contrast, the estimated purity of 324_A4 was 0.26 using our *H3F3A*-based method (Additional File 2: Figure S2E). Another popular tool purity analysis tool ASCAT [4] produced a purity estimate of 1.00 for 325_A4 with the ploidy estimated to be 4.7 (Additional File 2: Figure S2E). Comparison of purity estimate by H3 K27M founder mutation versus ABSOLUTE/ASCAT for all samples can be found in Additional File 3: Table S4.

***Using allelic imbalance to model evolution on chr17p loss***

Loss of chr17p often accompanies TP53 mutation to cause bi-allelic inactivation of TP53. In case 325, there are two TP53 mutations, R273H and R248W, which define the two major branches of the tumor evolutionary tree (Fig. 3A). Tumor regions 325_A3 and 325_A2 had the highest VAF for R273H (VAF 0.86) and R248W (VAF 0.63) mutations, respectively, which are considered representatives of these two branches. 325_A3 had chr17p LOH and a 1-copy gain of chr17q while 325_A2 had chr17p deletion with a diploid chr17q, and the two tumor regions share the same chr17p haplotype based on directional allelic imbalance analyzed by RECUR[5], indicating the potential for a common origin of chr17p loss. Therefore, we investigated two potential models: A) chr17p loss was a truncal variant and chr17p LOH at 325_A3 was due to subsequent amplification of entire chr17 which also resulted a copy gain on chr17q; B) chr17p LOH and chr17p loss were lineage-specific late events. Under model A, the *TP53* mutation would have occurred after chr17p loss while the order would be reversed under model B (Additional File 2: Figure S3A).

We tested these two models in sample 325_A2 to illustrate this process which involves using the *TP53* R248W VAF and BAF of heterozygous germline SNPs on chr17p. Sample 325_A2 has an estimated tumor purity of 0.78 (i.e. *t = 0.78*) and a BAF-adjusted copy value of 1.58 for chr17p suggesting that chr17p contains a mixture of 1-copy (haploid) and 2-copy (diploid) cells. As outlined in Additional File 2: Figure S3A, the cellular composition is different under these two models. Under model A which assumes that chr17p loss is a truncal variant present in all tumor cells, acquisition of the TP53 R248W mutation is expected to have been a secondary event affecting a fraction of haploid cells. This could have been followed by duplication of a subset of mutated haploid cells. Under model B, tumor cells first acquired the lineage-specific TP53 R248W mutation followed by loss of the wild-type chr17p in a subset of cells, resulting in haploid cells all containing the mutation.

Under both models, the tumor sample should contain a mixture of 1-copy haploid and 2-copy diploid cells. Using *f* to denote the fraction of haploid cells in the tumor, we estimated *f = 0.54* with copy number *cn = 1.58* and tumor purity *t = 0.78* according to the equation below:

$$cn=2\cdot\left( 1-t \right)+t\left[ f\cdot1+\left( 1-f \right)\cdot2 \right] = 2-t\cdot f$$

$$f=\frac{2-cn}{t}$$

Under Model A, all diploid tumor cells should harbor bi-allelic homozygous mutation while a fraction of haploid cells, denoted *g,* harbor the mutation. The VAF $(v)$of the R248W mutant allele can be calculated as outlined below:

$$v = \frac{t\left( gf+2(1-f) \right)}{c}=t\frac{2+f(g-2)}{c}$$

$$g=\frac{1}{f}\left( \frac{vc}{t}-2 \right)+2$$

Given the *v = 0.63* for *TP53* R248W mutation as measured by deep sequencing, the fraction can be estimated as: *g = 0.62*.

Under Model B, all haploid tumor cells harbor the mutant allele while a fraction of the diploid cells, denoted *g*, harbor the heterozygous R248W mutation. Consequently, the equation for the VAF of the R248W mutant allele is adjusted as follows:

$v = \frac{t\left( \left( 1-f \right)g+f \right)}{c}$

$$g= \frac{1}{1-f}\left( \frac{vc}{t}-f \right)$$

The resulting fraction of diploid cells with mutation, *g = 1.55*, rendering Model B invalid since the fraction of cells bearing an alteration cannot be above 1.0.

The origin of chr17p loss also affects the BAF of heterozygous germline SNPs on chr17p. Under Model A sample 325_A2 is comprised of a mixture of diploid normal cells, haploid tumor cells and diploid tumor cells with copy-neutral LOH. This is expected to result in two sets of BAFs, *b_1_* and *b_2_*, for SNPs with B-alleles on the deleted and retained haplotypes, respectively, defined as below:

$$b_{1}= \frac{1-t}{2-t\cdot f}$$

$$b_{2}= \frac{\left( 1-t \right)\cdot1+t\cdot\left( f\cdot1+\left( 1-f \right)\cdot2 \right)}{2-t\cdot f}=\frac{1+t-t\cdot f}{2-t\cdot f}$$

In Model B sample 325_A2 is comprised of diploid normal cells, diploid tumor cells and 1-copy tumor cells. The resulting BAFs, *b_1_* and *b_2_*, for SNPs with B-alleles on the deleted and retained haplotypes, respectively, are:

$$b_{1}= \frac{(1-t)\cdot1+t\cdot\left( 1-f \right)}{2-t\cdot f}=\frac{1-f\cdot t}{2-t\cdot f}$$

$$b_{2}= \frac{\left( 1-t \right)\cdot1+t\cdot\left( f\cdot1+\left( 1-f \right)\cdot1 \right)}{2-t\cdot f}=\frac{1}{2-t\cdot f}$$

The BAFs measured using tumor WES can be modeled, as done for copy number analysis, by Gaussian distributions. The results, presented in Additional File 2: Figure S3B, show that model B deviates significantly from the BAFs observed by WES.

Therefore, model A is selected as model B can be eliminated by the VAF of TP53 R248W as well as the BAF distribution of chr17p. Model A also fits the VAF of TP53 R273H mutation as well as BAF distribution of chromosome chr17 for tumor sample 325_A3, a representative region of the other branch (data not shown). We therefore placed chr17p loss as a truncal variant in the phylogenetic tree of case 325 (Fig. 3).

***Calculating cancer cell fractions (CCF) of a PDGFRA amplicon harboring PDGFRA^Y849C^ mutation***

To illustrate the process of calculating CCF of a mutation cluster accompanied by CNV, we used a mutational cluster comprised of FAM160A2^A239T^ CMA1^R58W^ and PDGFRA^Y849C^, identified by PyClone in case 311 (cluster B in Additional File 2: Figure S4A) as they co-occurred in three tumor regions 311_A3, 311_A4 and 311_A7. These three mutations are in genomic locations of different ploidy. The three mutation-positive regions have different anatomic locations: 311_A3 and 311_A4 are within the pons while 311_A7 is an extrapontine cerebellum sample. By applying the approaches outlined above we calculated the CCF of these mutations with the intermediate values presented in Additional File 2: Figure S4.

First, consider FAM160A2^A239T^ which is located on chr11 where diploidy accounted for 0.92-0.98 CCF across the three tumor regions. A model considering the presence of this mutation in non-diploid cells would have yielded *g* values > 1 (ranging 1.5-16.5), making this model invalid. By contrast, a model with this mutation present in diploid tumor cells is supported by valid *g* values across all three regions, resulting in CCFs of 0.37, 0.35 and 0.08 in 311_A3, 311_A4 and 311_A7, respectively.

Next, consider CMA1^R58W^, which is located on chr14 where 1-copy loss was found at CCFs of 0.48, 0.41 and 0.07 in 311_A3, 311_A4 and 311_A7, respectively. A model that considers this mutation occurs only in the 1-copy cells yields *g* values of 1.0, 0.95 and 1.2 for 311_A3, 311_A4 and 311_A7, respectively, indicating nearly all 1-copy tumor cells harbor this mutation. Although a *g* value of 1.2 in A7 exceeds 1, this is likely due to the imprecision in our measurement of the 1-copy deletion in this cell. If the CNV in A7 were changed from 1.95 to 1.935 (0.8% change), a valid g value of ≤1 would have been obtained. The resulting CCF values of CMA1^R58W^ are highly consistent with those of the diploid FAM160A2^A239T^ mutation. This example illustrates how minor deviation of an experimental measure may produce confounding results which need to be examined carefully.

The third mutation, PDGFRA^Y849C^, is located on chr4 where there is a copy number gain of the entire chromosome and a focal amplification of PDGFRA in all three regions (Additional File 2: Figure S4C). No allelic imbalance was detected on chr4 (Additional File 2: Figure S4B), indicating that the copy-gain was caused by bi-allelic duplication. By applying the CNV equation described above, we were able to calculate the CCF of cells with 4 copies of chr4 (*f_4_*) caused by bi-allelic duplication. We used the mean copy number across the entire chromosome for this estimate which resulted in *f_4_* values of 0.55, 0.43, and 0.84, higher than the CCF of the other two mutations in this cluster (i.e. FAM160A2^A239T^ CMA1^R58W^). This suggests that the chr4 duplication occurred earlier than acquisition of PDGFRA^Y849C^. Importantly, the omnipresence of chr4 duplication in all 7 tumor regions compared to the regional presence of PDGFRA^Y849C^ in three tumor regions corroborates the occurrence of chr4 duplication prior to PDGFRA^Y849C^ mutation.

Based on these data, we developed an evolutionary model with the following mutation acquisition order as outlined in Additional File 2: Figure S4A: 1) bi-allelic duplication of chr4; 2) acquisition of PDGFRA^Y849C^; and 3) focal amplification of PDGFRA. In step 3, the mutant allele is expected to be amplified because amplification of a wild-type allele can account for the focal CNV but would yield a VAF of PDGFRA^Y849C^ significantly lower than the empirical data (0.02 versus 0.10 at a read coverage of 3,103, p < 0.001 by Fisher’s exact test).

Under the above-mentioned model, each of the A3, A4 and A7 samples should be composed of normal diploid cells as well as four different types of tumor cells with the following chr4 characteristics: diploid, tetraploid, mutated tetraploid, and mutated tetraploid with local amplification. The copy number value under the proposed model can be expressed as follows:

$$c=2+t\left( 2f_{4}+f_{4}f_{P}gn \right)$$

$$g_{c}n= \frac{c-2-2tf_{4}}{tf_{P}}$$

Similarly, the VAF of PDGFRA^Y849C^ can be expressed as follows:

$$v= \frac{tf_{P}\left( 1+gn \right)}{c}$$

$$g_{vc}n= \frac{vc}{tf_{P}}-1$$

With these two sets of equations, we need to determine the values of *n* and *g*. However, because *g* is expected to be a fraction, the smallest number (*n*) of gained copies can be estimated by requiring *g ∊ <0;1>*. In A7 this number is about 7, and *g* values are approaching 1 indicating the subclone represented by this cluster all contains this PDGFRA amplicon which amplifies the PDGFRA^Y849C^ mutant allele by 7-fold; with an estimated CCF of 0.06. Assuming the same amplicon is also present in A3 and A4 we estimate that ~2% of this subclone also had PDGFRA amplification, which corresponds to a CCF of ~0.02.

***Refinement of phylogenetic trees by additional modeling***

In addition to the general process for constructing phylogenetic tree outlined methods, additional analysis was performed in selected cases to refine the structure. For cases 756 and 326 WES data were available for PDX/cell line models and additional clusters were manually added based on the mutational patterns in these models. When calculating CCFs for each mutation cluster, events such as focal amplification of *PDGFRA* required additional modelling to account for mutation multiplicity as described in preceding section. For a mutation cluster detected in multiple tumor regions, the tumor sample likely to produce the most accurate measure of CCF (high tumor purity and high VAF) was prioritized for lineage construction. Mutation clusters with ambiguous lineage are shown by dotted lines in figures.

Arm-level CNVs were incorporated into mutational clusters with matching CCFs; CNVs with a different BAF pattern (i.e. different retained haplotype) based on RECUR analysis were considered independent events. For CNV patterns with no matching mutational cluster, a CNV-only node was added to the appropriate lineage. In three cases (311, 312 and 324), a subgroup of truncal variants had CCF <1.0 in a subset of the tumor samples. These subclonal truncal variants, referred to as “pervasive” mutations in a previous study on colorectal cancer[6], were placed in the same branch as the clonal truncal variants but labeled distinctly.

***Evaluation of truncal variants with low CCF***

Truncal variants with CCF <1, detected in 3 cases (311, 312, 324), were further assessed for the possibility of reduced VAF due to deletion of the mutant allele, which was reported in a prior study on multi-region sequencing of lung cancer[5]. We were able to rule out this possibility by comparing the CCF of the deletion with the VAFs of somatic mutations. An example is the *TP53* in-frame indel R174_E180>R detected in all five tumor regions of case 324, with VAF ranging from 0.25 to 0.62. All tumor samples in this case also harbored chr17p loss estimated to have a CCF of 1.0 (clonal) based on modeling of BAF and tumor purity (estimated from the *H3F3A* K27M mutation). These data indicate that the *TP53* mutation was acquired after the chr17p loss, as a clonal chr17p deletion of the *TP53* mutant allele would have led to a VAF of 0. The *TP53* mutation was estimated to have CCFs of 0.43, 0.79, 0.73, 0.52 and 0.70 in A1-A5 regions, confirming that its low VAFs were due to subclonal presence rather than loss of a mutant allele.

**Statistical analysis on assessment of residual tumor content in normal brain samples**

To determine whether an anatomically normal brain samples contained residual tumor, we first assessed the presence of the founder mutation *H3F3A* K27M using the deep sequencing data generated from normal samples of the 9 patients that had *H3F3A* K27M. The two patients with *HIST1H3B* K27M mutation were not analyzed for the following reasons: 1) normal sample of case 160 was from blood; and thus not suitable for this analysis; 2) deep sequencing data of the normal sample from case 312 shown presence of truncal variants of tumor samples from a different case, indicating a technical contamination.

To distinguish low abundance mutations from sequencing errors in deep sequencing data in the normal brain samples, we employed an approach previously developed for assessing minimal residual disease (MRD) in serial bone marrow samples of acute lymphoblastic leukemias[7] which involves two-steps: 1) determining the read count of reference and mutant allele by using sequencing reads that passed error suppression by the CleanDeepSeq algorithm [8]; and 2) using samples expected to be wild-type, i.e. no target mutation detected in tumor samples, to calculate the background error rate which is in turn used for calculating the statistical significance of the mutant allele fraction in the index samples using binomial distributions. A sample was called mutation-positive if the FDR-corrected p < 0.001. The mutation-negative samples for the analysis of residual *H3F3A* K27M were from the two cases (i.e. 312 and 160) that harbor *HIST1H3B* K27M mutation. In addition to single-variant analysis we also calculated the probability of the presence of a mutational cluster (e.g. all truncal variants in the case of *H3F3A* K27M founder mutation) as a result of co-occurrences of sequencing errors by performing a Fisher’s combined probability test on all constituent variants. This analysis was also used for determining clonal composition of residual tumor present in normal samples.

Four normal samples, two from case 311 (G2 and G3) and two from case 326 (G3 and G4), were found to have residual H3F3A K27M mutations with VAF ranging from 0.01 to 0.1 (Additional File 2: Figure S5). The results obtained from the mutation cluster representing truncal variants were consistent with those from the founder mutation H3F3A K27M analysis (Additional File 2: Figure S5). In case 311 mutations defining subclones B and C were both present in normal sample G3 while only clone B mutations were present in normal sample G2. In case 326, G3 and G4 samples were positive for both the truncal variants and mutations defining clone E.

1. **Supplementary Table legends**

**Supplementary TableS1. Demographical, histopathological characteristics and treatment history of all samples used for molecular profiling.**

Col A: identifier for the whole-exome sequencing data.

Col B: identifier for each patient which is based on the last 3-digit of the sequence_id.

Col C: sample ID defined by patient ID plus region number. Tumor and normal samples are appended with _G and _A, respectively.

Col D: is the sample tumor or normal.

Col E: position provided by pathologist by using fractions to present clockwise positions within pons. For example: 0.5 is 6'clock. The 12 o’clock position (abutting the cerebellum) is shown on the bottom in figures

Col F: age of onset

Col G: male (M) or female (F).

Col H: tumor purity estimated based on founder mutation H3 K27M. N/A is marked for normal samples.

Col I: duration of treatment (in days)

Col J: enrollment of patients in the newly diagnosed DIPG (Stratum A) or progressive DIPG (Stratum B) as defined in NCT01393912 (reference 16).

Col K: cycle of treatment (reference 16)

Col L: crenolanib dose used for the treatment (reference 16)

**Supplementary TableS2. Tumor regions occupied by tumor cells descending from multiple lineages.** A lineage is defined as a branch established by an immediate “child” of the founder clone.

Col A: tumor sample ID defined by patient ID plus tumor region number.

Col B: number of clonal lineages detected in the tumor sample.

Col C: subclones (labeled by their mutational cluster ID shown in the main and/or supplementary figures for each patient) descending from the predominant lineage in the tumor region.

Col D: CCF of subclones defined in Col C.

Col E: CCF of the ancestral clone. This refers the clone marked as * in the clonal decomposition figure.

Col F: Combined CCF of col D & E. 1-ColF is the most conserved estimate of the CCF of subclones descending from a second lineage.

**Supplementary TableS3**

Col A= DIPG tumor samples

Col B = Purity estimated by ABSOLUTE software

Col C= Purity estimation based on H3K27M

Col D= Purity estimation based on ASCAT

**Supplementary TableS4**

Col A= DIPG tumor samples

Col B= Purity estimation based on H3K27M

Col C= Number of clones in tumor samples

Col D= Mutation/Mb

Col E = newly diagnosed DIPG (Stratum A) or progressive DIPG (Stratum B)

**Reference:**

1. Edmonson MN, Zhang J, Yan C, Finney RP, Meerzaman DM, Buetow KH. Bambino: A variant detector and alignment viewer for next-generation sequencing data in the SAM/BAM format. Bioinformatics. 2011;

2. Rusch M, Nakitandwe J, Shurtleff S, Newman S, Zhang Z, Edmonson MN, et al. Clinical cancer genomic profiling by three-platform sequencing of whole genome, whole exome and transcriptome. Nat Commun. 2018;9.

3. Carter SL, Cibulskis K, Helman E, McKenna A, Shen H, Zack T, et al. Absolute quantification of somatic DNA alterations in human cancer. Nat Biotechnol. 2012;30:413–21.

4. Van Loo P, Nordgard SH, Lingjærde OC, Russnes HG, Rye IH, Sun W, et al. Allele-specific copy number analysis of tumors. Proc Natl Acad Sci [Internet]. 2010;107:16910 LP – 16915. Available from: http://www.pnas.org/content/107/39/16910.abstract

5. Jakubek YA, San Lucas FA, Scheet P. Directional allelic imbalance profiling and visualization from multi-sample data with RECUR. Bioinformatics. 2019;35:2300–2.

6. Sottoriva A, Kang H, Ma Z, Graham TA, Salomon MP, Zhao J, et al. A Big Bang model of human colorectal tumor growth. Nat Genet. 2015;47:209–16.

7. Li B, Brady SW, Ma X, Shen S, Zhang Y, Li Y, et al. Therapy-induced mutations drive the genomic landscape of relapsed acute lymphoblastic leukemia. Blood. 2020;135:41–55.

8. Ma X, Shao Y, Tian L, Flasch DA, Mulder HL, Edmonson MN, et al. Analysis of error profiles in deep next-generation sequencing data. Genome Biol. 2019;20:50.
