# Supplementary material for: Convergent evolution and multi-wave clonal invasion in H3 K27-altered diffuse midline gliomas treated with a PDGFR inhibitor
Source: Acta Neuropathol Commun. 2022 May 31;10:80. doi: 10.1186/s40478-022-01381-0 (PMC9153212; doi:10.1186/s40478-022-01381-0)
Supplement: Supplementary file 2 — Additional file2. Supplementary figures. [file 40478_2022_1381_MOESM2_ESM.pdf]

Figure S1

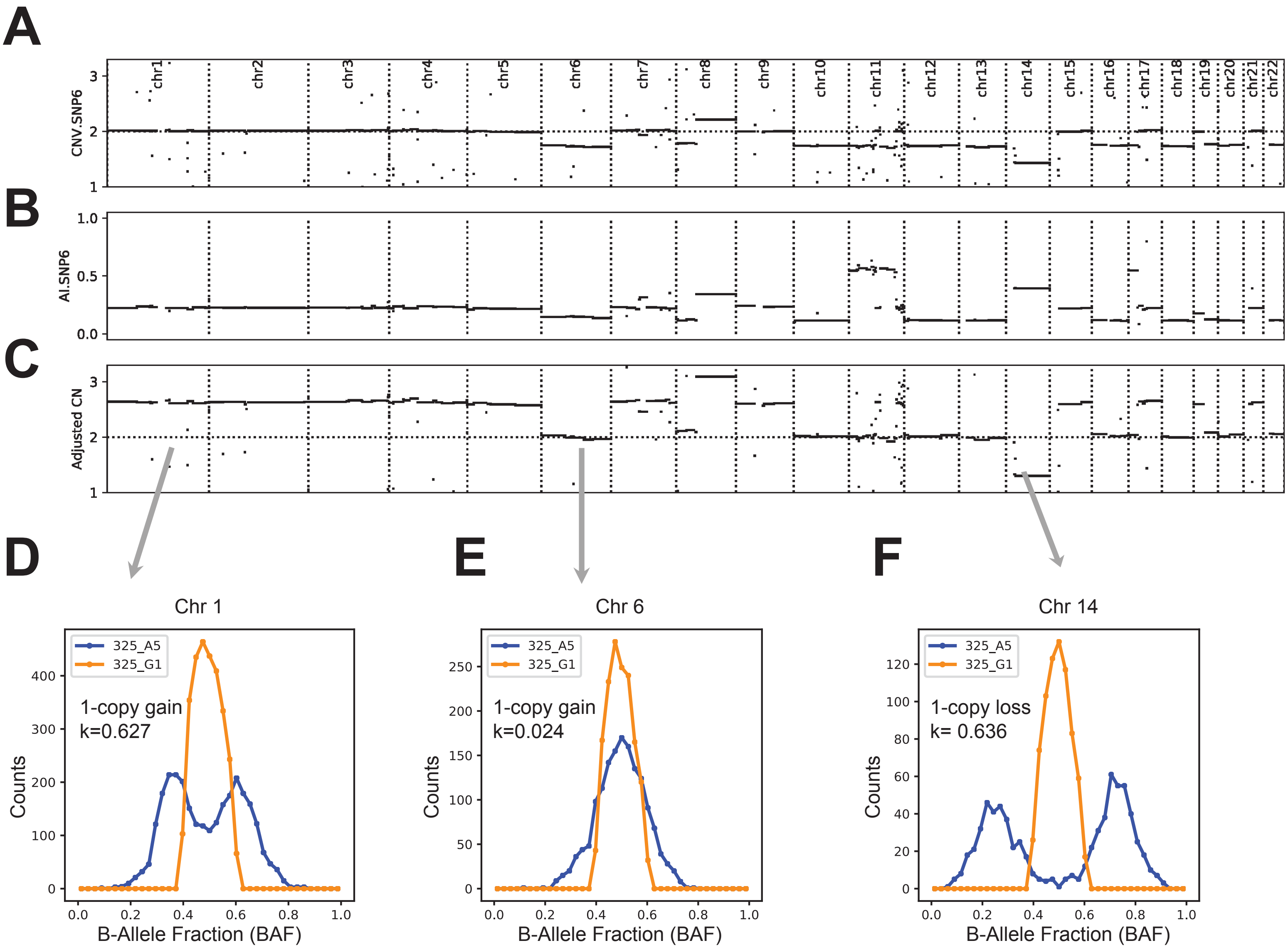

Figure S1: An example of BAF-adjusted somatic copy number in a tumor sample.
This example is based on somatic CNV in tumor sample 326_A5. Genome-wide SNP array analysis of somatic CNV (**A**) and allelic imbalance (AI) (**B**) shows inconsistency between the ploidy and AI (e.g. multiple 2-copy regions with AI >0). (**C**) Adjusted copy number profile based on BAF of germline heterozygous SNPs in tumor WES. BAF distribution in tumor (blue line) and normal (orange line) for representative chromosomes are shown in (**D**)-(**E**). (**D**) 1-copy gain of chr1 with an estimated high cellular prevalence (*k*) of 0.627 in contrast to the diploid status projected by SNP array. (**E**) Low cellular prevalence ($k = 0.02$) of 1-copy gain on chr6 indicating vast majority of the cells are diploid in contrast to the CN loss projected by SNP array. (**F**) 1-copy loss on chr14 present with an estimated to have a high cellular prevalence of 0.636.

Figure S2

A

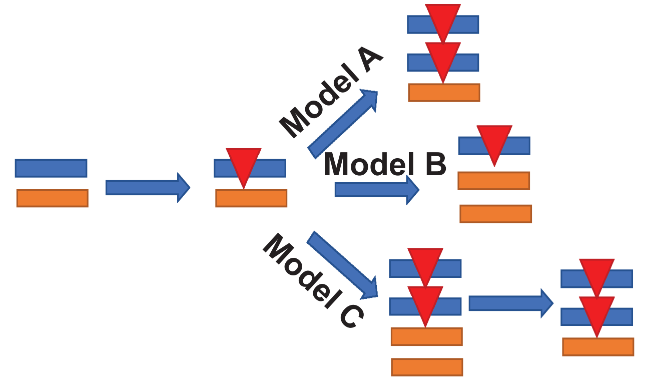

B

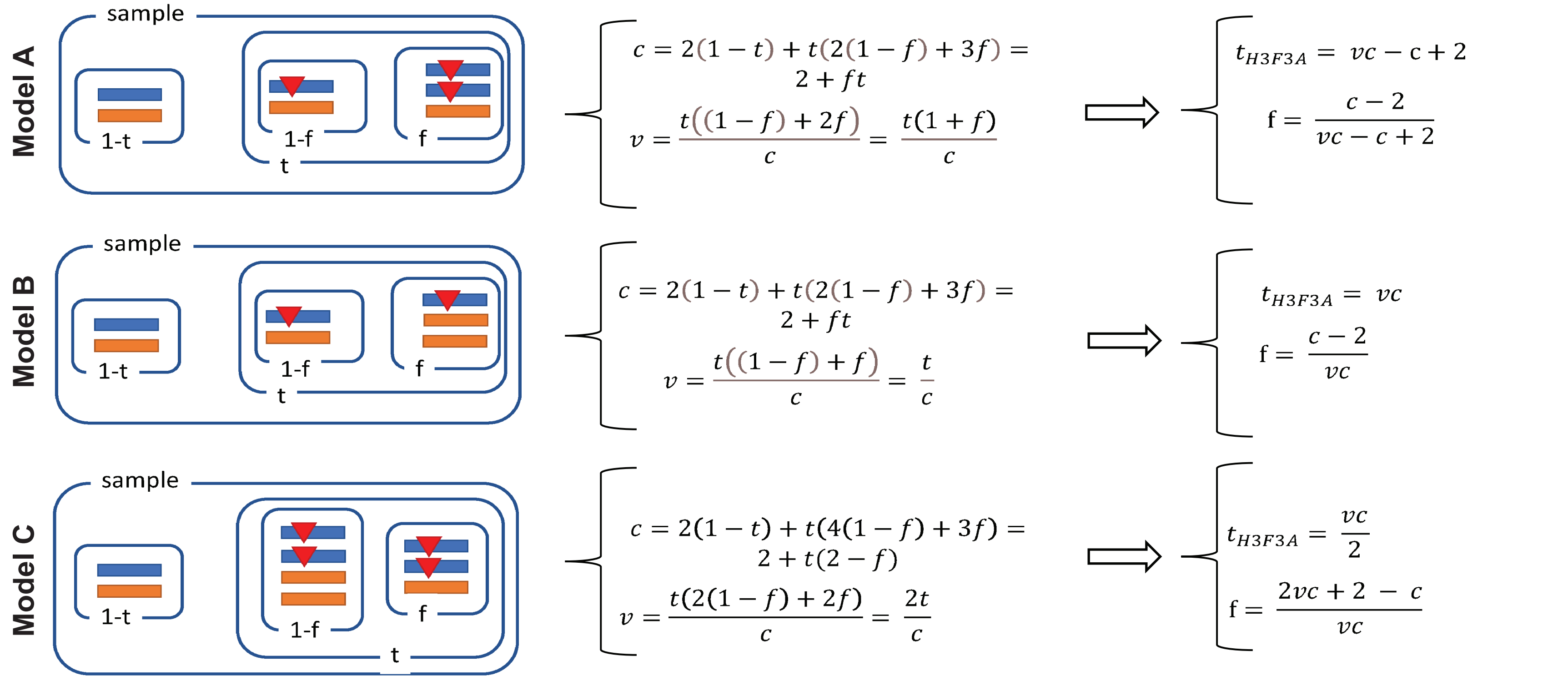

C

Sample	VAF	CN	Model A		Model B		Model C	
			t	f	t	f	t	f
325_A2	0.396	2.02	0.78	0.03	0.80	0.11	0.40	1.94
325_A3	0.567	2.80	0.79	1.03	1.59	3.98	0.79	0.98
325_A4	0.184	2.13	0.26	0.51	0.39	1.53	0.20	1.32
325_A5	0.512	2.67	0.70	0.99	1.37	3.47	0.68	1.02

D

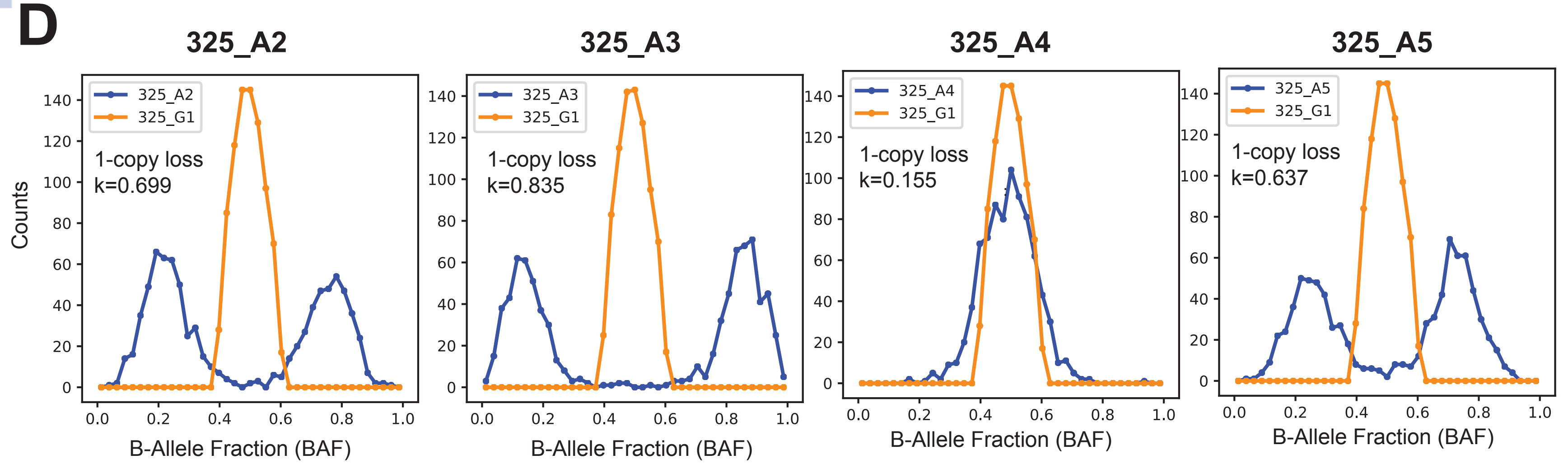

E

Sample	ABSOLUTE Purity estimation	ABSOLUTE Ploidy estimate	ASCAT Purity estimate	ASCAT Ploidy estimate	t_{H3F3A}	k (chr14 loss)
325_A2	0.6	3.79	0.37	4.73	0.78	0.7
325_A3	0.44	3.41	0.60	2.73	0.79	0.84
325_A4	0.66	1.95	1.00	4.7	0.26	0.16 (approximate)
325_A5	0.37	3.47	0.67	2.83	0.70	0.64

Figure S2: An example of tumor purity assessment based on a founder mutation.
The four tumor regions profiled by both WES and SNP array from case 325 were assessed for their purity based on the cellular prevalence of the founder mutation H3F3A^{K27M} which is accompanied by a subclonal 1-copy gain of chr1. (A) There are three possible models, labeled Model A, Model B and Model C, describing acquisition of 1-copy gain after H3F3A mutation (labeled by a red triangle). (B) Tumor purity (*t*) and tumor cell clonal composition resulting from each model will affect both observed copy number (*c*) and the VAF of the H3F3A mutation (*v*). Tumor purity estimation and triploid cell fraction (B on the right) yields values (C) that indicates that only Model A is feasible for all 4 tumor samples. Estimated cellular prevalence (*k*) of 1-copy loss of chr14, a truncal variant present in all four tumor regions using the BAF-adjusted CNV method (D), is compared to tumor purity estimated based on H3F3A mutation and calculated by ABSOLUTE analysis on SNP array data (E)

Figure S3

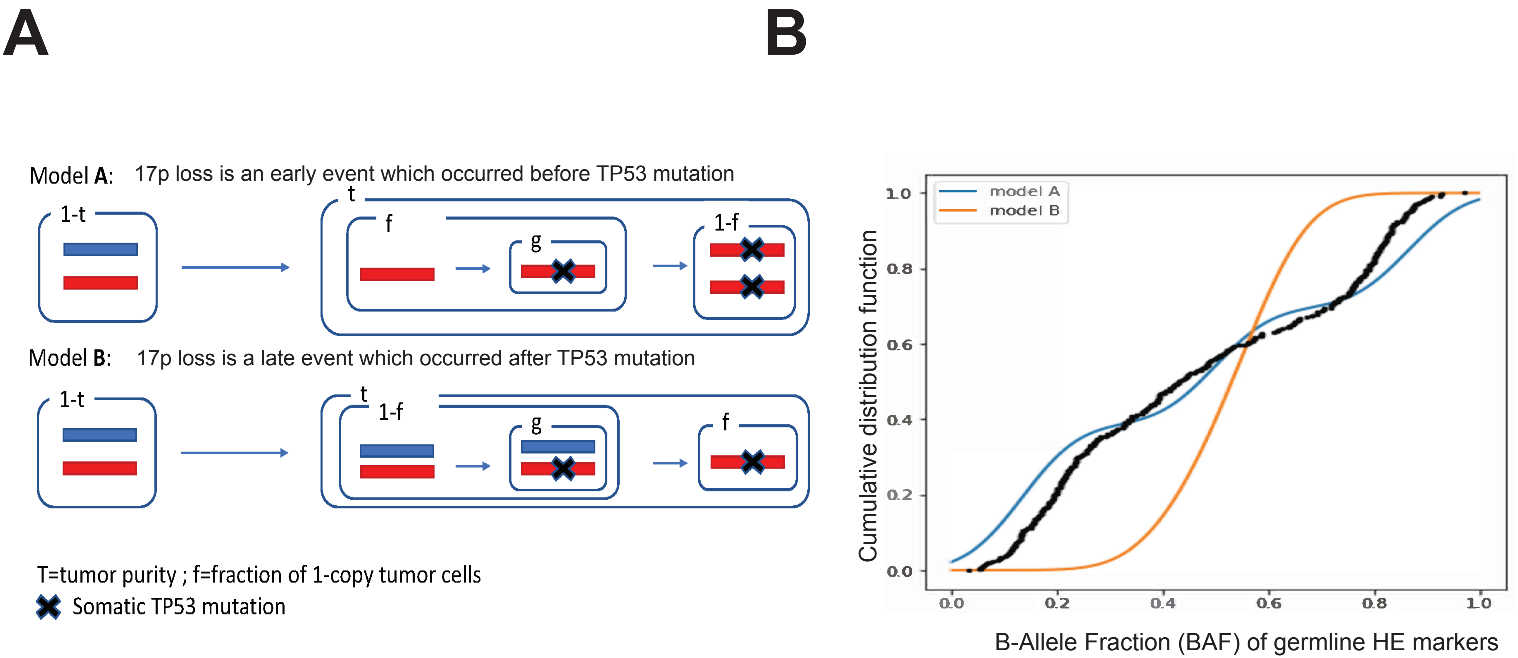

Figure S3: Modeling the timing of chr17p loss in case 325
Loss of chr17p often accompanies TP53 mutation to cause bi-allelic loss of TP53 function. Case 325 has two distinct TP53 mutations in different tumor regions and we modeled whether chr17p loss could have occurred before (Model A) or after (Model B) acquisition of TP53 mutation in tumor sample 325_A2 (**A**). Composition of tumor and normal cells based on evolutionary trajectory defined in Model A and Model B in sample 325_A2. (**B**) Cumulative distribution plot of germline heterozygous SNP BAFs on chr17p in 325_A2 (black dots) compared to hypothetical distributions that are based on model A (blue) and model B (orange).

Figure S4

A. Evolution of PDGFRA

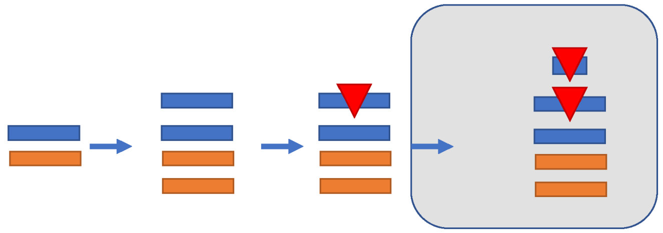

B. PDGFRA model

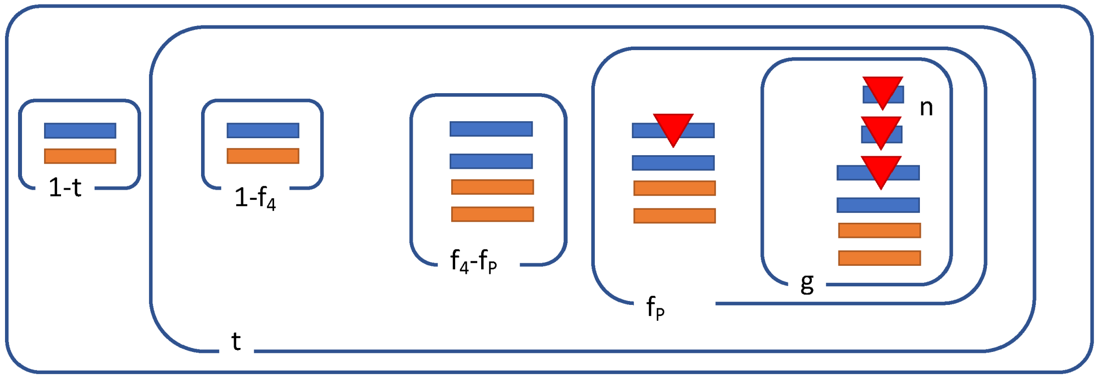

C

FAM160A2^{A239T}

Sample	VAF	CN	t	f_2	g_1/g_3	g_2	CCF
311_A3	0.156	1.98	0.83	0.98	16.5	0.38	0.37
311_A4	0.133	2.06	0.8	0.92	4.4	0.38	0.35
311_A7	0.03	1.96	0.8	0.95	1.5	0.08	0.08

CMA1^{R58W}

Sample	VAF	CN	t	f_1	g_1	CCF
311_A3	0.250	1.60	0.83	0.48	1.0	0.48
311_A4	0.185	1.68	0.8	0.41	0.95	0.39
311_A7	0.03	1.95	0.8	0.07	1.2	0.08

PDGFRA^{Y849C}

Sample	VAF	^MCN	t	f_4	^LCN	f_P	n*	g_c	g_{vc}	CCF ^{amp}
311_A3	0.164	2.9	0.83	0.545	3.02	0.48	7	0.05	0.08	0.028
311_A4	0.149	2.69	0.8	0.434	2.87	0.39	7	0.08	0.05	0.02
311_A7	0.117	3.35	0.8	0.844	3.92	0.08	7	1.27	0.88	0.07

Figure S4: Cancer cell fraction calculations for PDGFRAY^{849C} amplicon in case 311.
This data presented here correspond to the example described in the section of "Calculating cancer cell fractions of somatic mutation clusters and CNVs" in Materials and Methods. PDGFRAY^{849C} was grouped in mutational cluster B which is present A3, A4 and A7 as shown in Figure S4A. (A) A model of *PDGFRA* mutation and copy evolution where blue and orange bars represent chromosomal haplotypes, and the red triangle shows a PDGFRAY^{849C} mutation acquired after chr4 bi-allelic duplication. (B) Tumor sample is comprised of normal diploid cells, tumor diploid cells, tetraploid cells, mutated tetraploid cells, and mutated tetraploid cells with local amplification. Fractions of these cell types can be determined by: tumor purity (t), fraction of tetraploid cells in the tumor (f_4) estimated from mean copy number (MCN), fraction of a clone harboring a mutation (f_P) estimated from other mutations composing the same clone, and fraction of tetraploid mutated cells that bear the local copy gain (g) as well as number of gained copies (n). Number of copies gained was assumed to be the same across the samples, and its value is the smallest that renders this model feasible for all samples (C, bottom). Estimated CCFs of cluster B based on a mutation in the diploid region (FAM160A2) and one in a region with 1-copy loss (CMA1) shown at the top enabled estimate of the n in sample 325_A7.

Figure S5

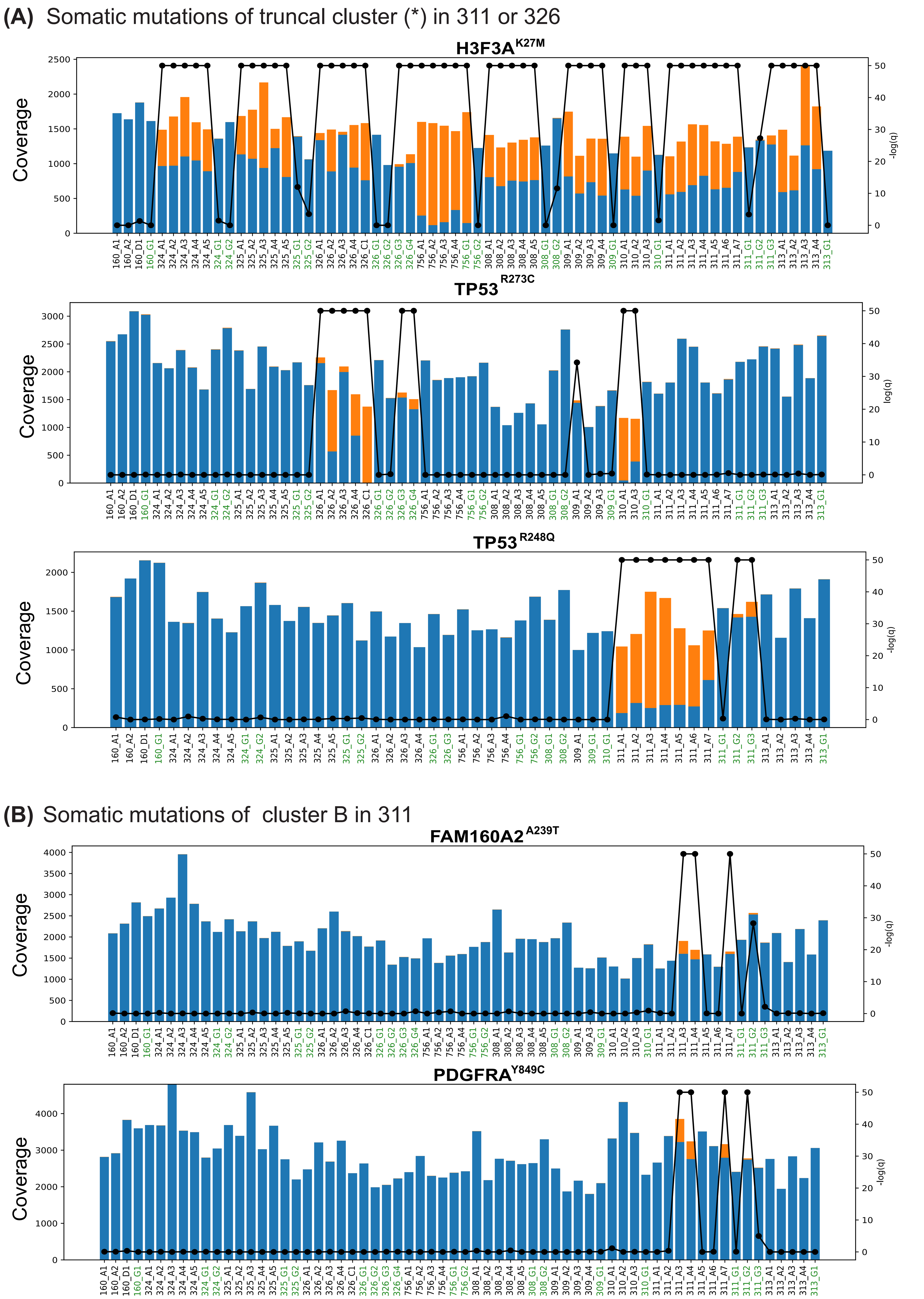

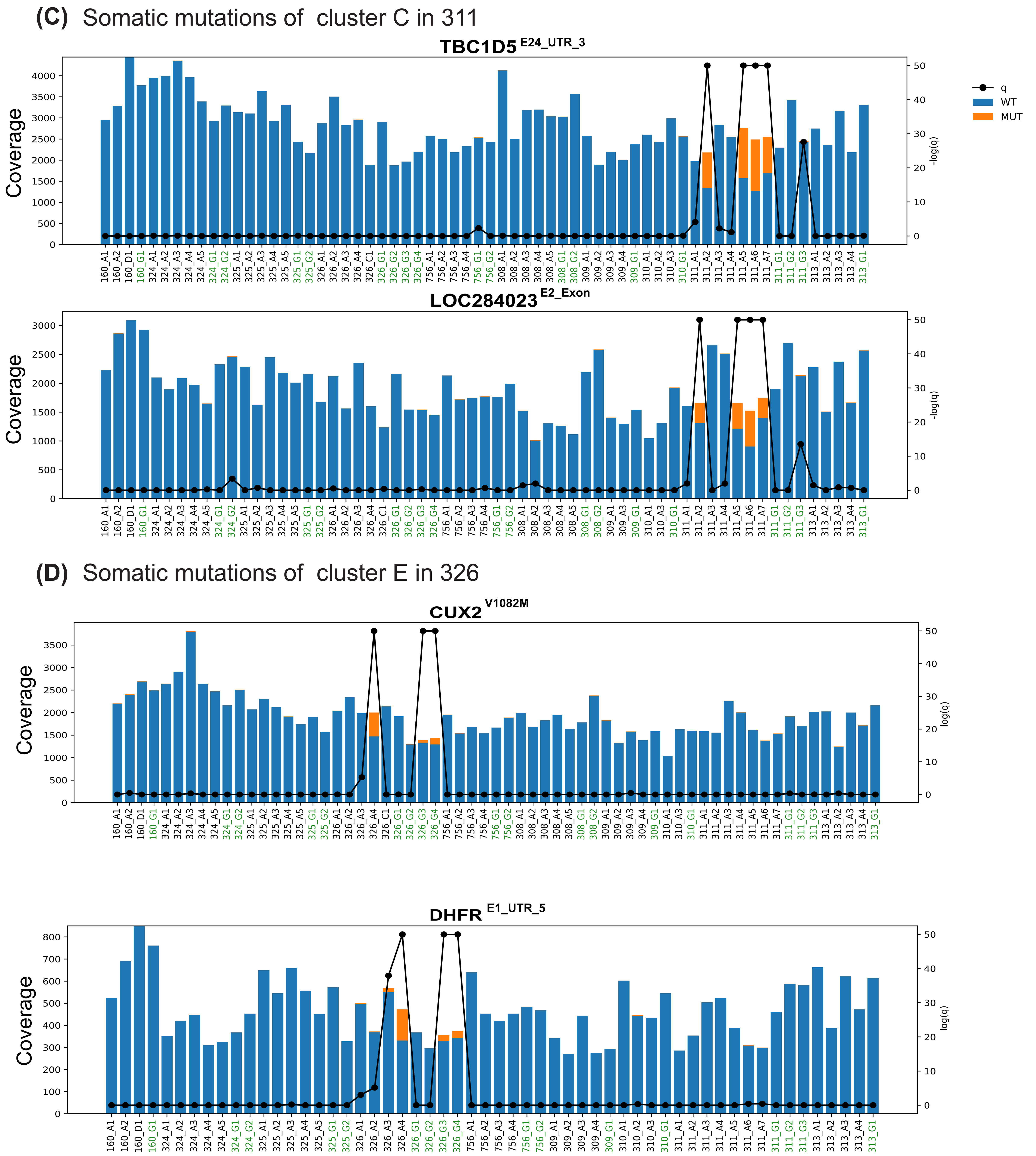

(E) Significance of mutation clusters in 311 (B and C) and 326 (D)

	Truncal variants		Cluster B		Cluster C	
Sample	χ^2	p-value	χ^2	p-value	χ^2	p-value
G1	7.36	0.29	0.13	0.99	0	1
G2	260.62	2.2e-53	159.63	1.73e-33	0	1
G3	∞	~0	14.22	6.6e-3	82.64	5.85e-17

	Truncal variants		Cluster E	
Sample	χ^2	p-value	χ^2	p-value
G1	0	1	0.05	0.99
G2	0.45	0.9778	~0	~1
G3	331.8	1.493e-17	1148.37	2.26e-238
G4	1115.79	2.86e-240	∞	~0

Figure S5: Residual tumor in normal brain samples of case 311 and 326.
Bar plots (**A**) showing read counts of reference (blue) and mutant alleles in representative somatic mutations forming truncal mutations H3K27M in both patients while TP53 R273C and R248Q are truncal variants for 326 and 311 respectively. Bar plots (**B and C**) showing read counts of reference (blue) and mutant alleles in representative somatic mutations forming cluster B mutations, and cluster C mutations, respectively in 311. Bar plots (**D**) showing read counts of reference (blue) and mutant alleles in representative somatic mutations forming cluster E mutations in 326. FDR corrected probability (*q*) calculated based on binomial model of sequencing errors are shown as connected black dots. (**E**) Fisher's combined probability and corresponding χ^2 test of detecting clusters in samples labeled as germline in 311 and 326 respectively.

FigureS6

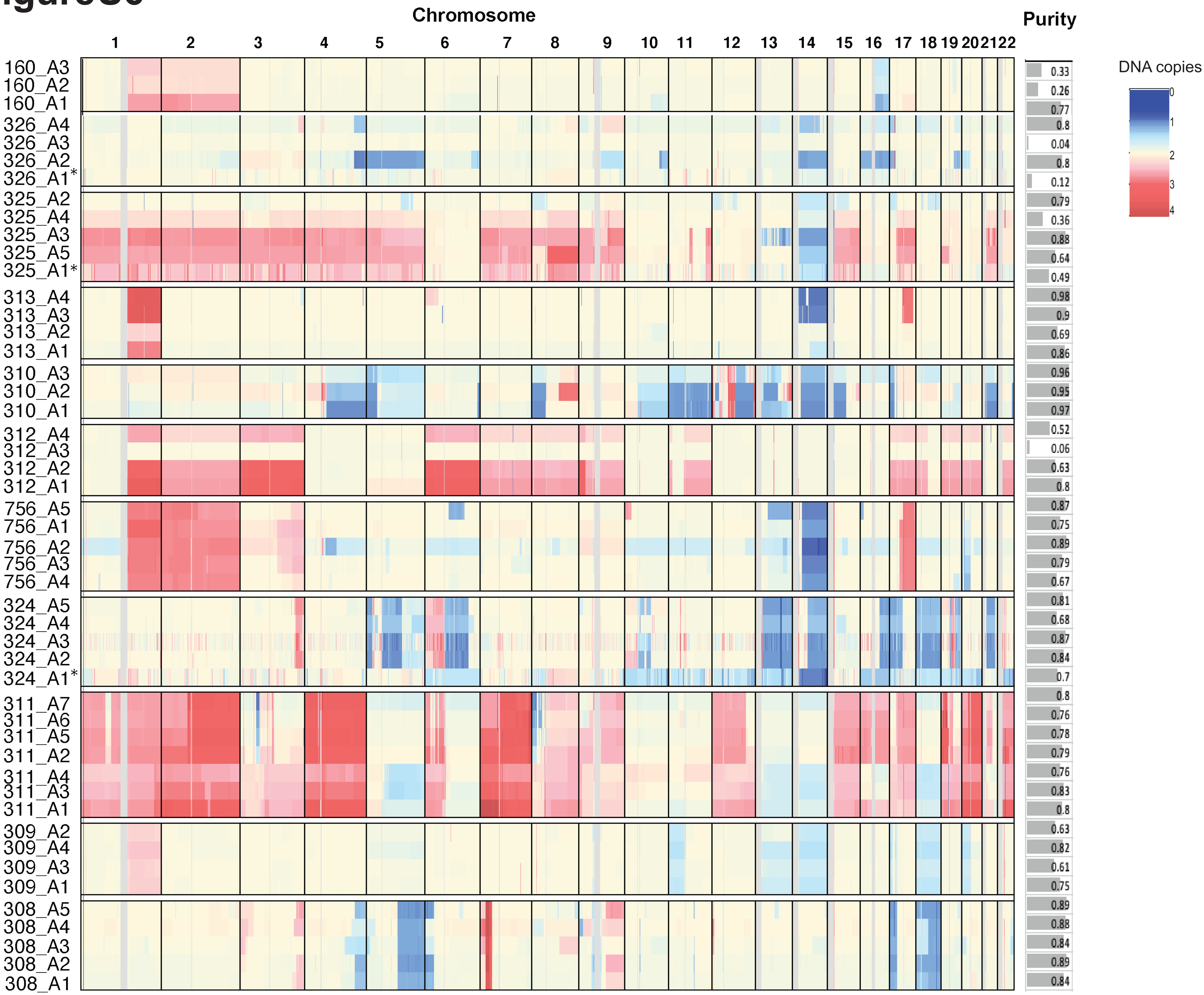

Figure S6: Somatic CNV plot by SNP array

Somatic CNV plot by SNP array or exome CNV (marked by *) with blue color indicating copy loss and red indicating copy gain with purity for all samples.

Figure S7

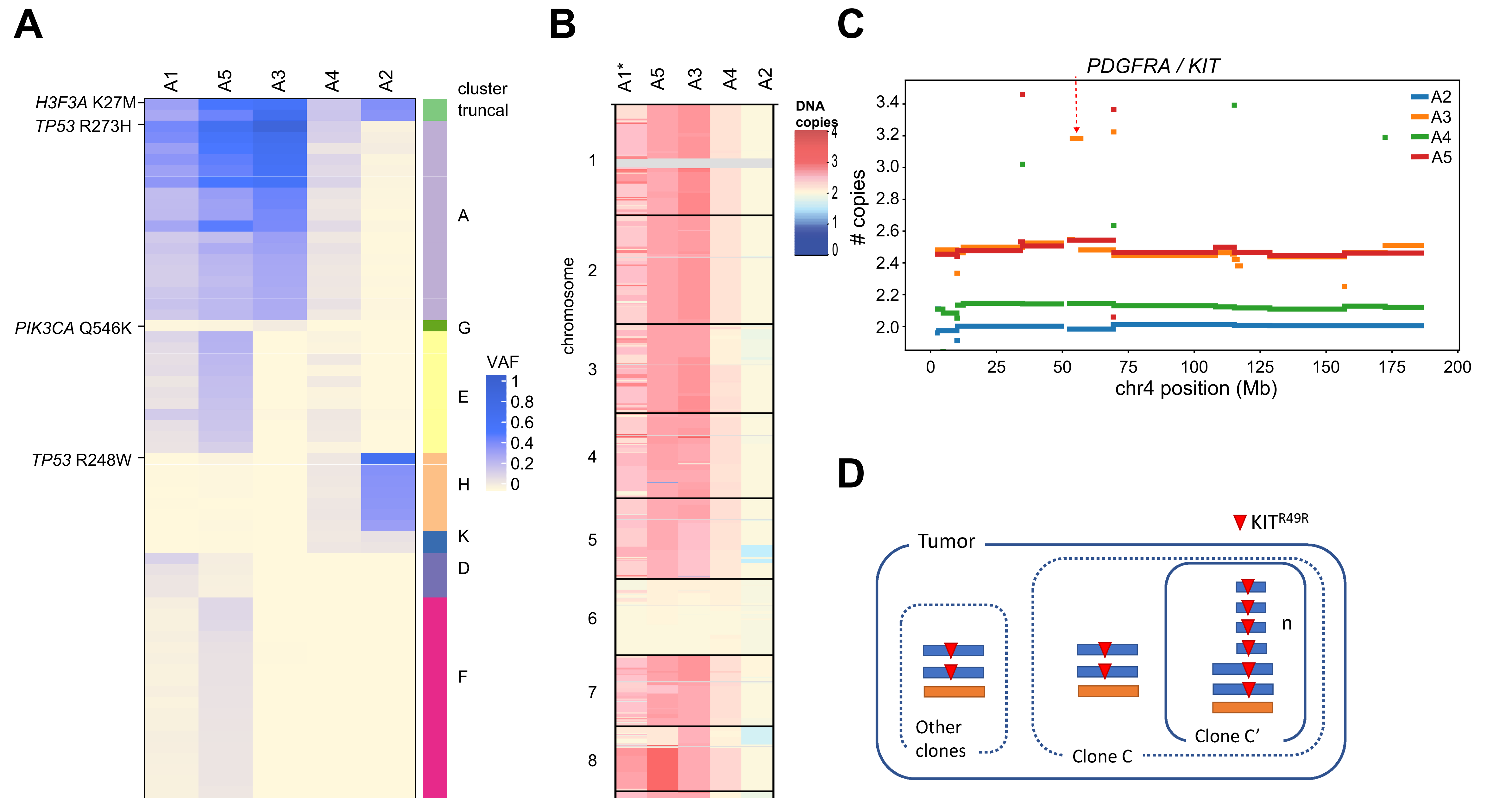

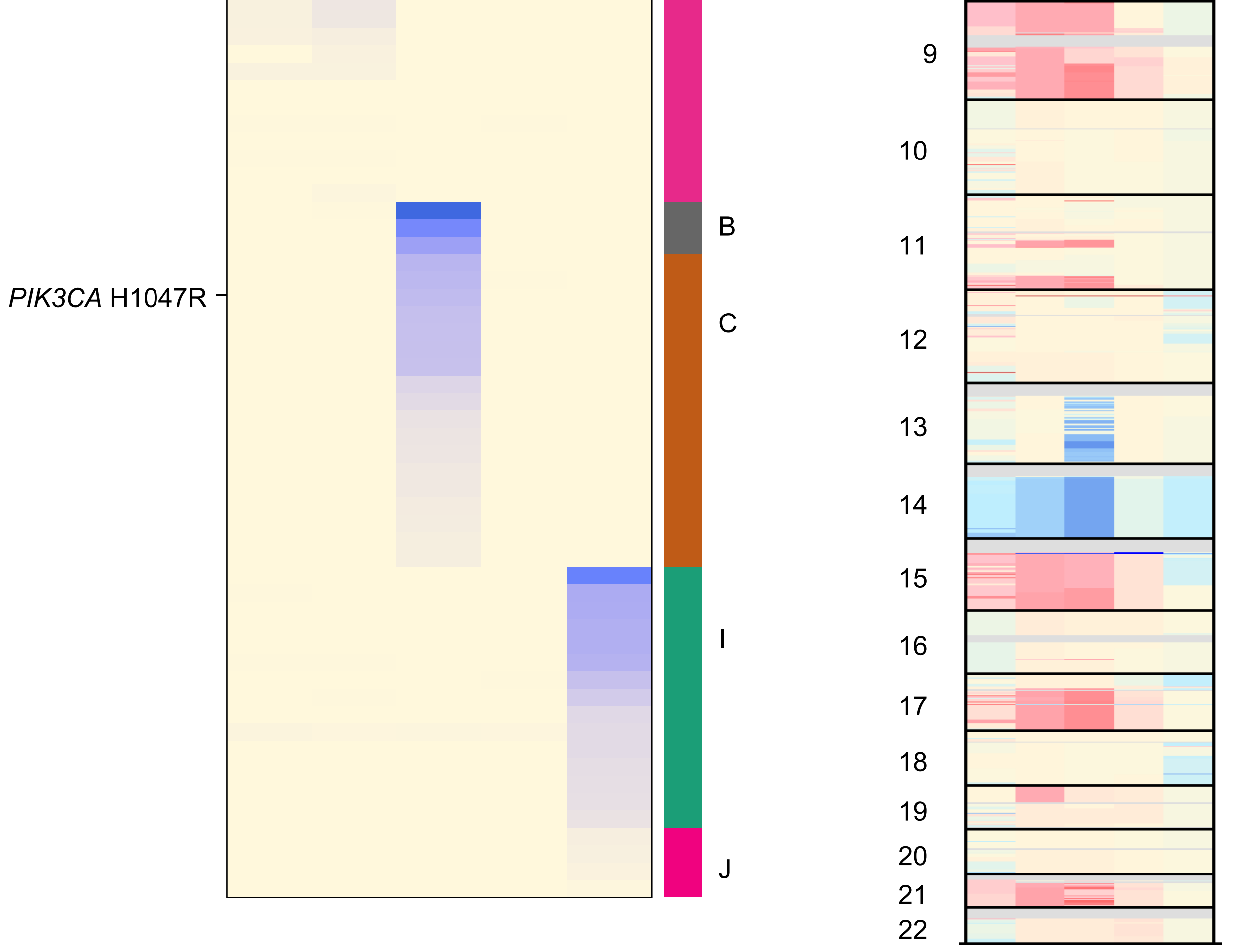

Sample	Copy number at PDGFRA/KIT	VAF of KIT	CCF_C	n	g_{VAF}	g_{CN}	$CCF_{C'}$
325_A3	3.18	0.66	0.68	4	0.16	0.22	0.18

Figure S7: Multi-region mutational clusters and copy number alterations in patient 325.
The data content matches those shown in Figure 3. **(A)** Clusters of somatic SNVs/indels from the 5 profiled tumor regions. The tumor samples are ordered based on the similarity of clustering pattern. Each cluster is marked by an alphabetic ID and a distinct color at the right. The cluster ID is also used to label the corresponding node on the phylogenetic tree and subclone ID shown in Figure 3. VAF of each mutation is indicated by the blue shade and selective driver mutations are labeled. **(B)** Somatic CNV plot by SNP array (A2-A5) or exome CNV (A1, marked by *) with blue color indicating copy loss and red indicating copy gain. The tumor samples are displayed in the same order as **(A)**. **(C)** Chromosome 4 CNV plot of A2-A5, highlighting a focal amplification of PDGFRA/KIT present only in 325_A3. **(D)** Calculating CCF and copy number of a focal amplification involving PDGFRA/KIT in 325_A3. The amplicon, which is unique to 325_A3, is expected to be acquired in clone C (see phylogenetic tree at **Figure 3),** a 325_A3-specific subclone defined by cluster C in panel **A**. All tumor cells in 325_A3 harbor a 1-copy gain of chr4 on the haplotype containing the founder mutation KIT^{R49R}. Top: a model of tumor cell composition which includes clone C along with its descent clone C' which contains the amplicon estimated to have 4-copy gain as well as tumor cells from other clones. Haplotypes are depicted with blue (duplicated) and orange (not duplicated) bars. Bottom: a table with the numerical values describing the model. CCF_C refers to CCF of cluster C; the g_{VAF} and g_{CN} refers to the fraction of 3-copy tumor cells that contain the focal amplification calculated from the VAF of KIT^{R49R} and copy number at PDGFRA/KIT, respectively. $CCF_{C'}$ refers to the final CCF estimated for clone C'.

Figure S8

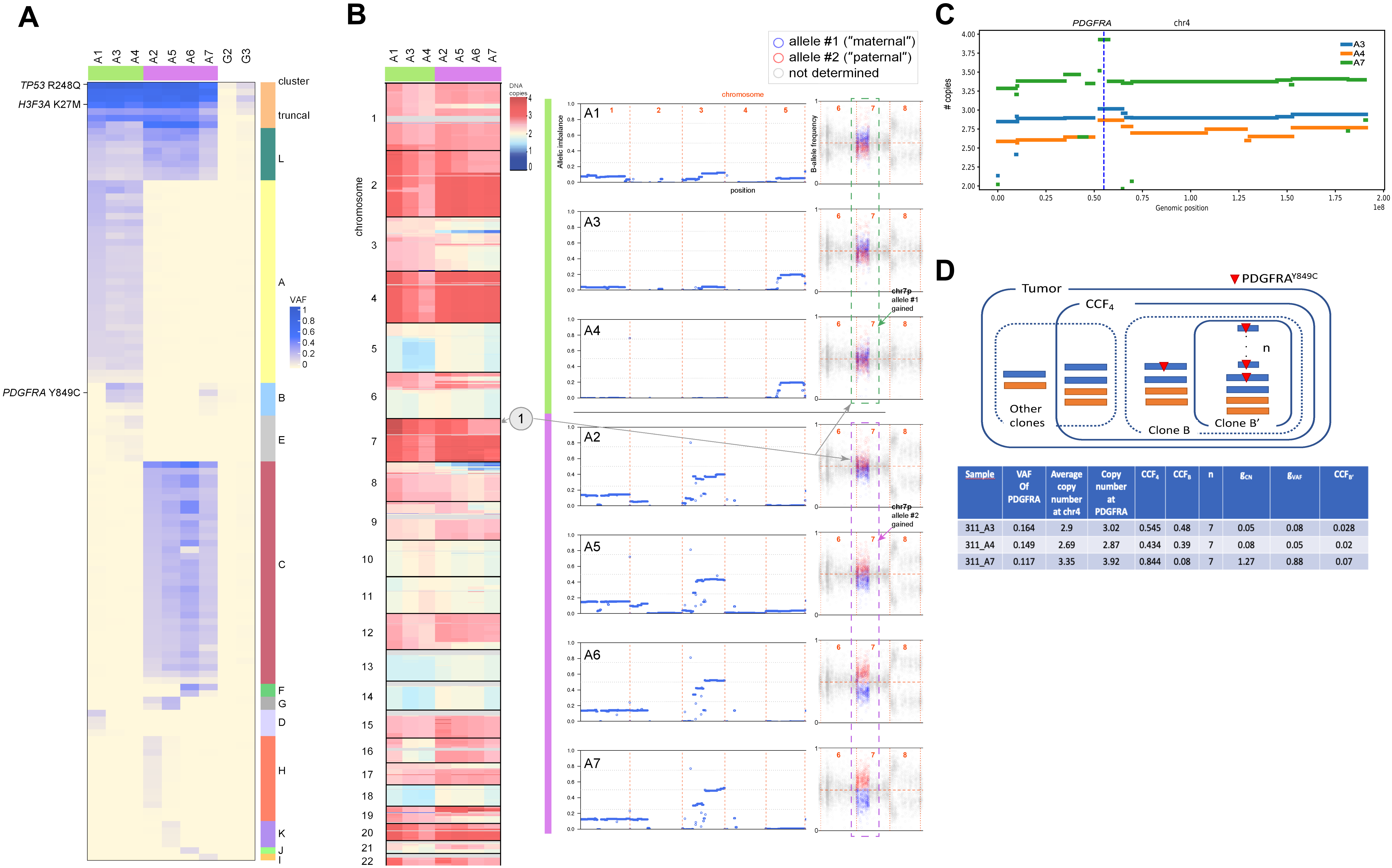

Sample	VAF Of PDGFRA	Average copy number at chr4	Copy number at PDGFRA	CCF_4	CCF_B	n	g_{CN}	g_{VAF}	$CCF_{B'}$
311_A3	0.164	2.9	3.02	0.545	0.48	7	0.05	0.08	0.028
311_A4	0.149	2.69	2.87	0.434	0.39	7	0.08	0.05	0.02
311_A7	0.117	3.35	3.92	0.844	0.08	7	1.27	0.88	0.07

Figure S8: Multi-region mutational clusters and copy number alterations in patient 311.

The data content matches those shown in Figure 4. **(A)** Clusters of somatic SNVs/indels from the 7 profiled tumor regions. Two normal samples (G2 and G3) containing residual tumor are also included. **(B)** Left, somatic CNV plot. Right, allelic imbalance (AI) plot on a subset of the chromosomes including chr4 where the lack of AI cab be explained by a bi-allelic duplication. Inset 1 shown differential maternal and paternal allele copy gains are highlighted in chromosome 7. **(C)** Chromosome 4 CNV plot of three tumors (A3, A4 and A7) that harbor full-chromosome copy gain and a focal PDGFRA amplification. **(D)** Calculating CCF and copy number of PDGFRA amplicon. The amplicon is expected to be acquired in clone B (see phylogenetic tree at **Figure 4**) as cluster B mutations (panel **A**) shown the same prevalence as the PDGFRA amplicon. Top: a model of tumor cell composition which includes clone B along with its descent clone B' containing the amplicon estimated to have 7-copy gain (marked by n) as well as tumor cells from other clones that have a mixture of 2-copy and 4-copy chr4. $PDGFRA^{Y849C}$ was acquired after bi-allelic duplication of chr4. Bottom: a table with the numerical values describing the model in samples A3, A4 and A7. CCF_4 and CCF_B refers to CCFs of chr4 b-allelic duplication and clone B, respectively; the g_{VAF} and g_{CN} refer to the fraction of 4-copy tumor cells that contain the focal amplification calculated from the VAF of $PDGFRA^{Y849C}$ and copy number at PDGFRA, respectively. $CCF_{B'}$ refers to the final CCF estimated for clone B'

Figure S9

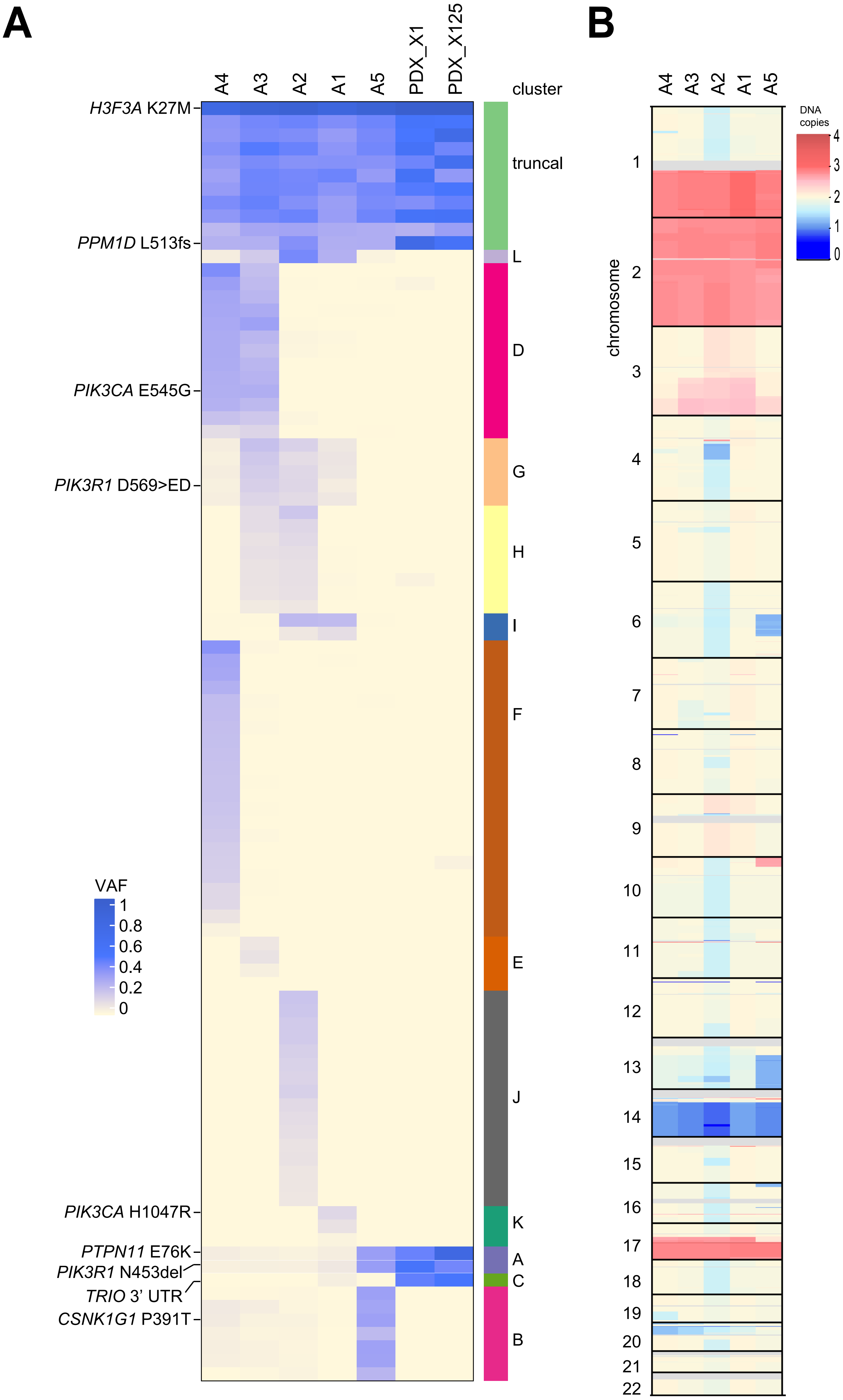

Figure S9: Extensive PI3K convergent evolution and uncertain invasion path between the pons and an extrapontine region in patient 756

(A) Clusters of somatic SNVs/indels from the 5 profiled tumor regions and two patient derived xenograft samples (PDX) derived from A1. Two PDX samples were used to deconvolute clusters A, C, and B. Cluster L has a singleton mutation at 17p present in all five tumor regions with varying CCFs (range 0.04-0.90) and was not incorporated in phylogenetic tree construction. **(B)** Somatic CNV plot showing only tumor sample

Figure S10

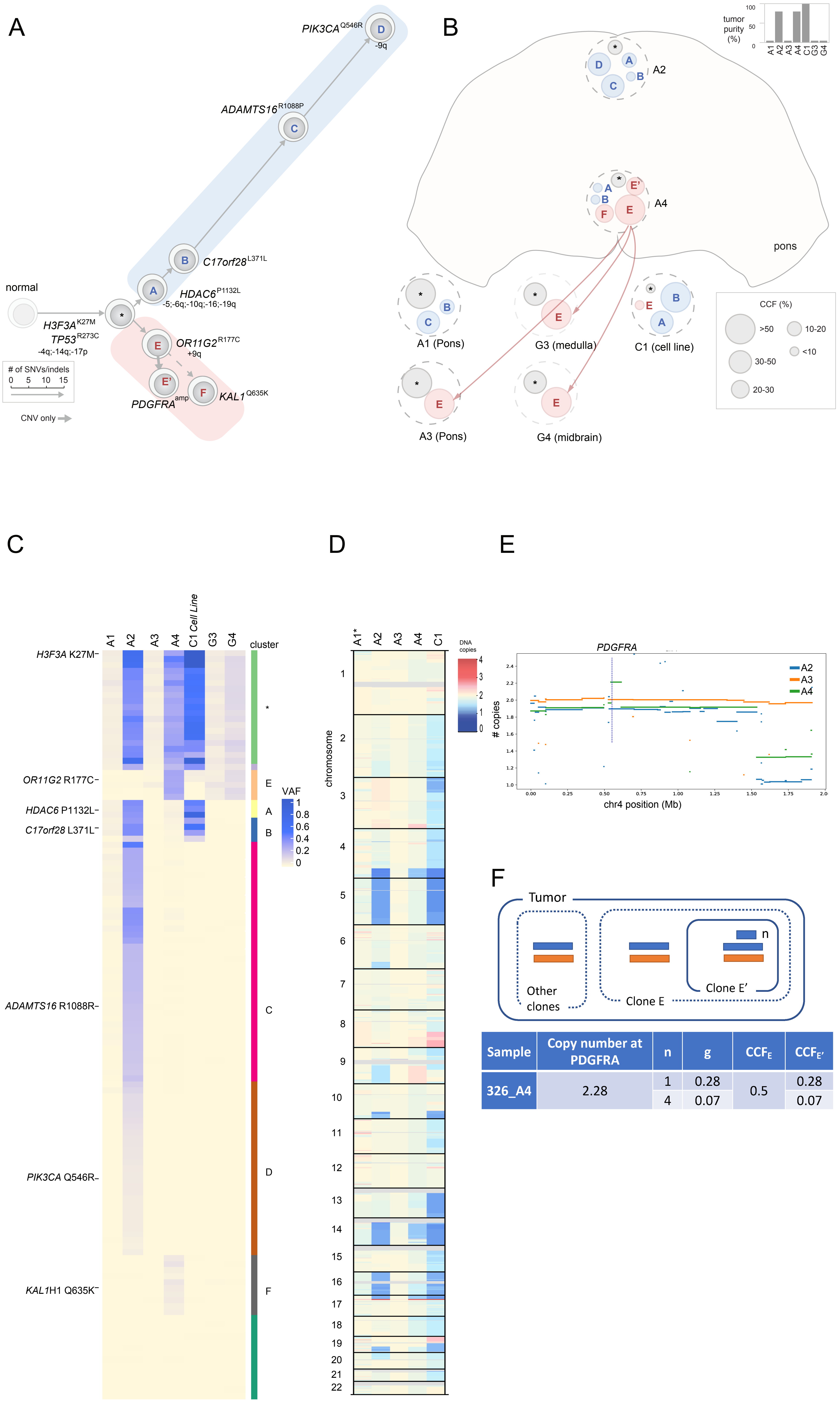

Figure S10: Extrapontine invasion to medulla and midbrain detected in normal brain samples in patient 326

The Panels A and B are drawn in same style as Figure 3. **(A)** An evolutionary tree constructed from two high-purity tumor samples (A2 and A4) and a cell line (C1) derived from a low-purity tumor sample (A1). **(B)** Spatial position and clonal composition of four tumor samples, a cell line (C1), and two histologically normal extrapontine samples that contained low-abundance tumor cells likely due to extrapontine invasion of clone E or E' of the short (pink) branch from A4 to medulla (G3) and midbrain (G4). **(C)** Clusters of somatic SNVs/indels from the 4 profiled tumor regions along with two normal samples (G3 and G4) containing residual tumor and a cell line (C1) derived from A1. **(D)** Somatic CNV plot by SNP array (A2-A4) or exome CNV (A1, marked by *) and cell line C1. **(E)** Copy number plot of chromosome 4 highlighting subclonal *PDGFRA* amplification in A4. **(F)** Calculating CCF and copy number of PDGFRA amplicon in A3. The amplicon, which is unique to A3, is expected to be acquired in clone E or its descendant F, a A3-specific branch defined by cluster E/F in panel **A**. Top: a model of tumor cell composition which includes clone E along with its descent clone E' containing the PDGFRA amplicon. Below: the number of focal copy-gain at PDGFRA can have multiple solutions and CCF derived from an estimate of 1 and 4 copy gain is shown in the table. This resulted in ambiguous placement of the amplicon either as a descent of E (for n=1) or could be a descent of either E or F (for n=4) as the CCF of clone F is <0.2.

Figure S11

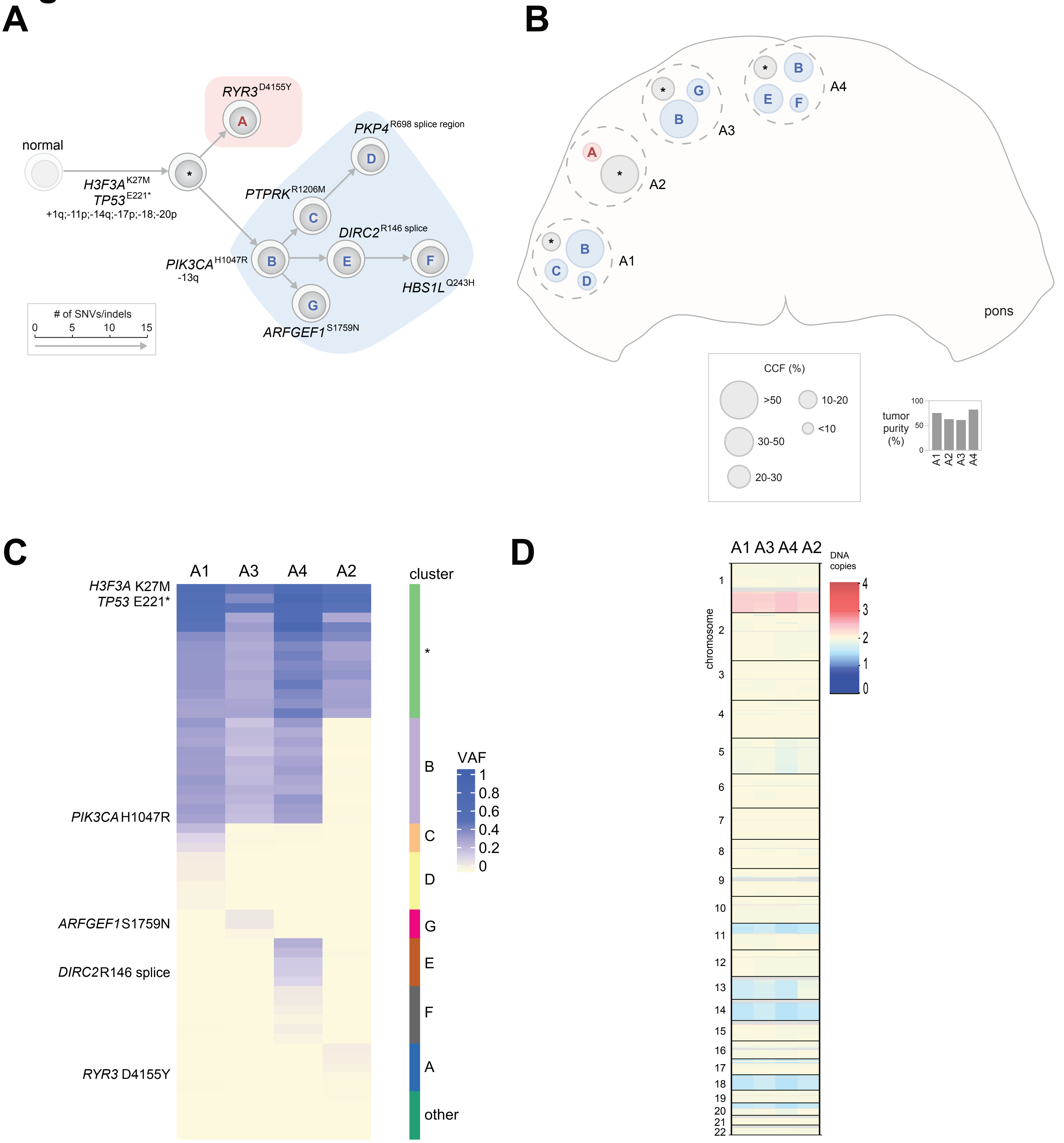

Figure S11: Phylogenetic tree and clonal composition of case 309.
The two panels **(A-B)** are drawn using the same style as Figure 3. No convergent evolution was detected. **(A)** Evolutionary tree consists of long blue branch seeded by a PIK3CA^{H1047R} mutation and very short pink branch; spatial position and clonal composition of three tumors are shown in **(B)**. **(C)** Heatmap of somatic SNVs/indels with selected variants marked. **(D)** Somatic CNV plot of four tumor samples.

Figure S12

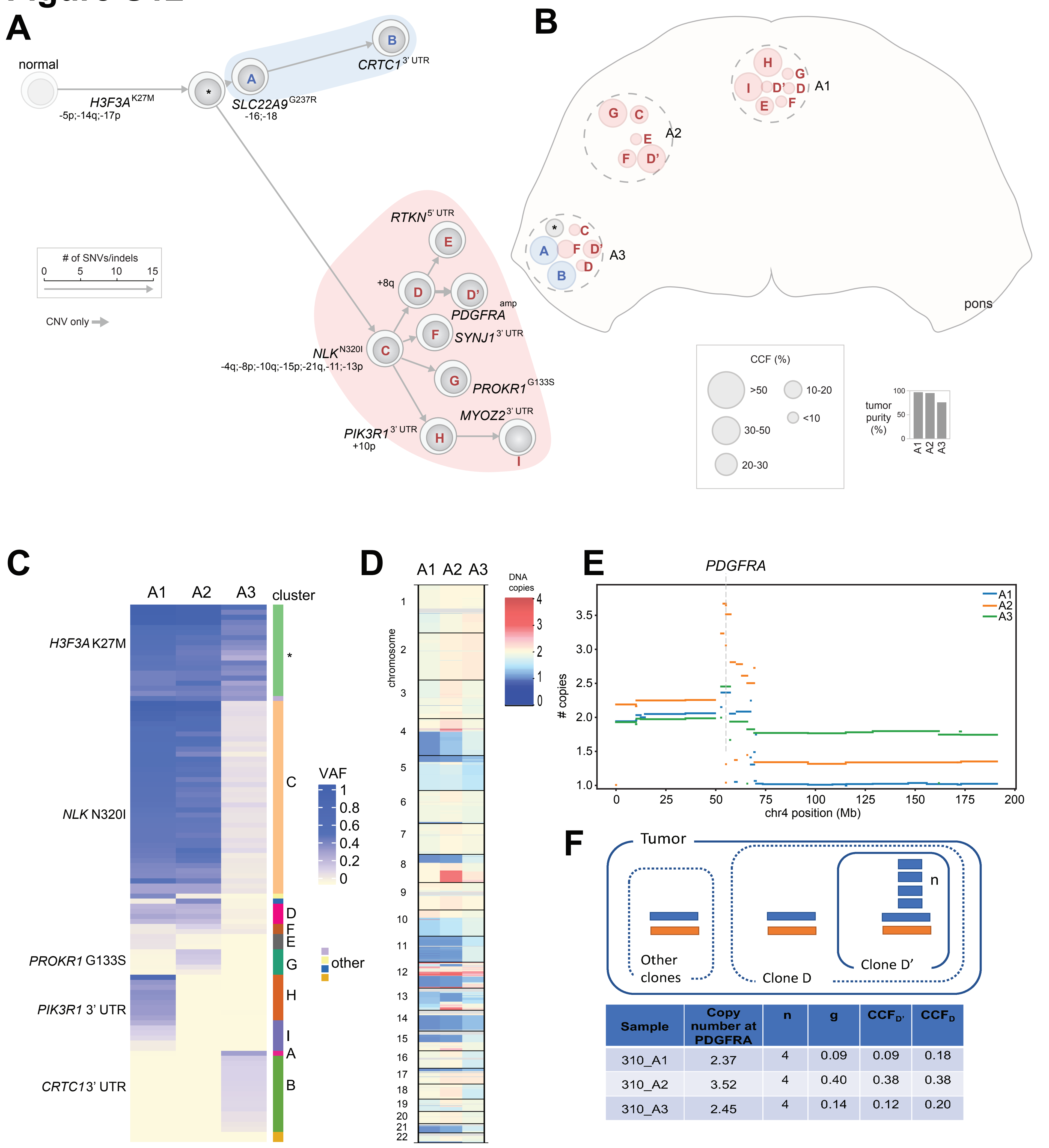

Sample	Copy number at PDGFRA	n	g	$CCF_{D'}$	CCF_{D}
310_A1	2.37	4	0.09	0.09	0.18
310_A2	3.52	4	0.40	0.38	0.38
310_A3	2.45	4	0.14	0.12	0.20

Figure S12: Phylogenetic tree and clonal composition of case 310

PDGFRA focal amplification was present in all three tumor regions but at differ in their amplification level. (A) Evolutionary tree with a long pink branch bearing *PDGFRA* amplification and a short blue branch. *PDGFRA* amplification is a descendant of clone D and labeled as clone D'. (B) Spatial position and clonal composition of three tumors showing A3 has admixed lineages. (C) Heatmap showing somatic SNVs and indels. (D) Somatic CNV in all tumor samples. (E) Absolute copy number (y-axis) in each sample on chromosome 4 (x-axis shows chromosome coordinates), with the *PDGFRA* gene indicated by a vertical gray line. *PDGFRA* was amplified in all samples. (F) A model of tumor cell composition showing copy number aberrations of *PDGFRA* where n is the copy gains of the *PDGFRA* amplicon (estimated to have four-copy gain) and $CCF_{D'}$ is the CCF of *PDGFRA*-amplified cells. The table below shown the CCFs of cells with *PDGFRA* copy number aberrations in all three samples

Figure S13

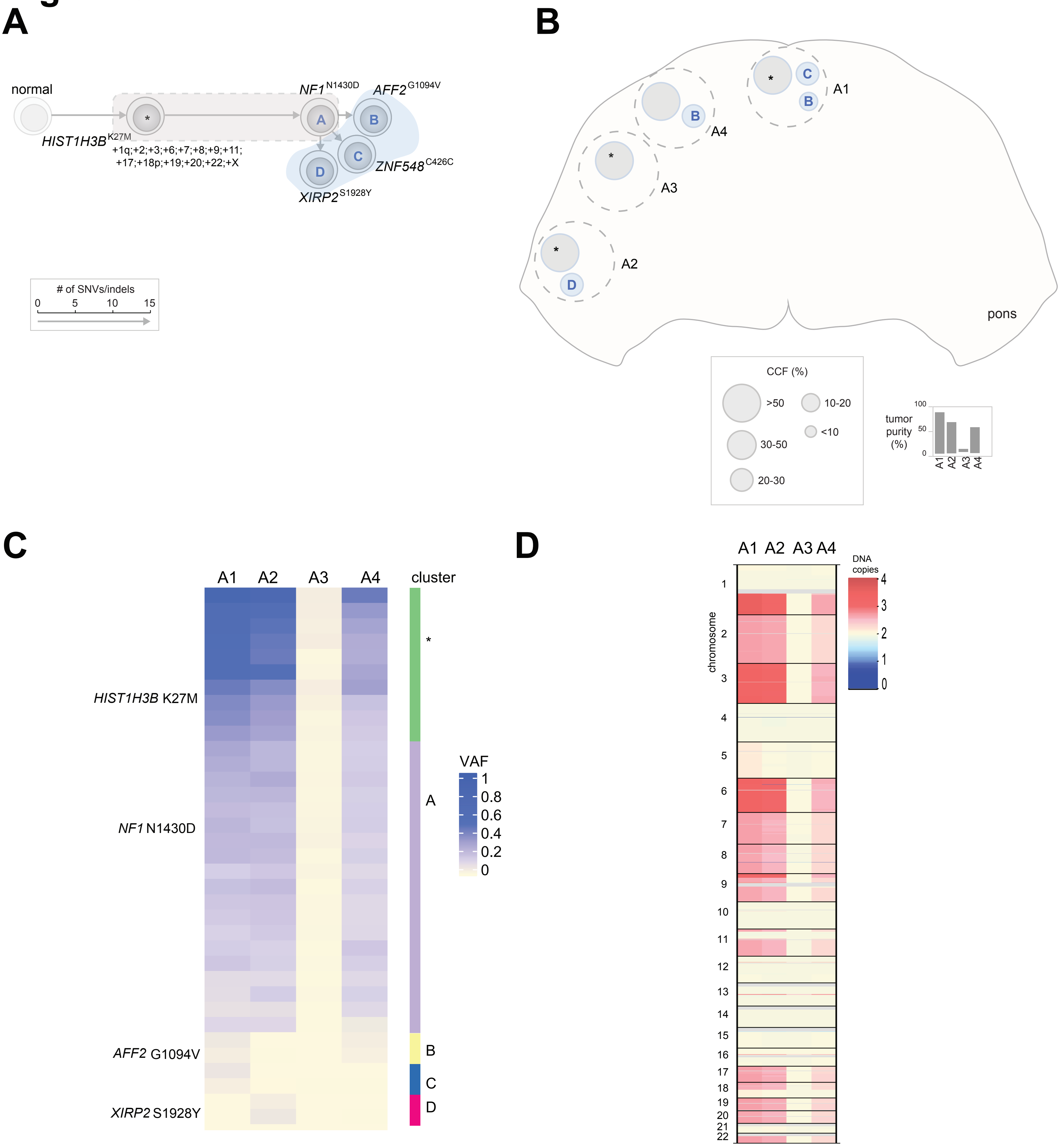

Figure S13: Phylogenetic tree and clonal composition of case 312

This case has a H3.1 H3K27M mutation and is the only one that has a single branch. **(A)** An evolutionary tree constructed from three high-purity tumor samples (A1, A2 and A4). Truncal variants consist of a cluster representing the founder clone (*) and cluster A including a NF1 mutation with varying CCFs in different tumor regions which are outlined with a dotted line; the descending nodes can arise either from the founder clone (*) or clone A **(B)** Spatial positions and clonal composition of each sample, with tumor purity indicated below. **(C)** Heatmap showing somatic SNVs and indels. **(D)** Somatic CNV in all four tumor samples.

Figure S14

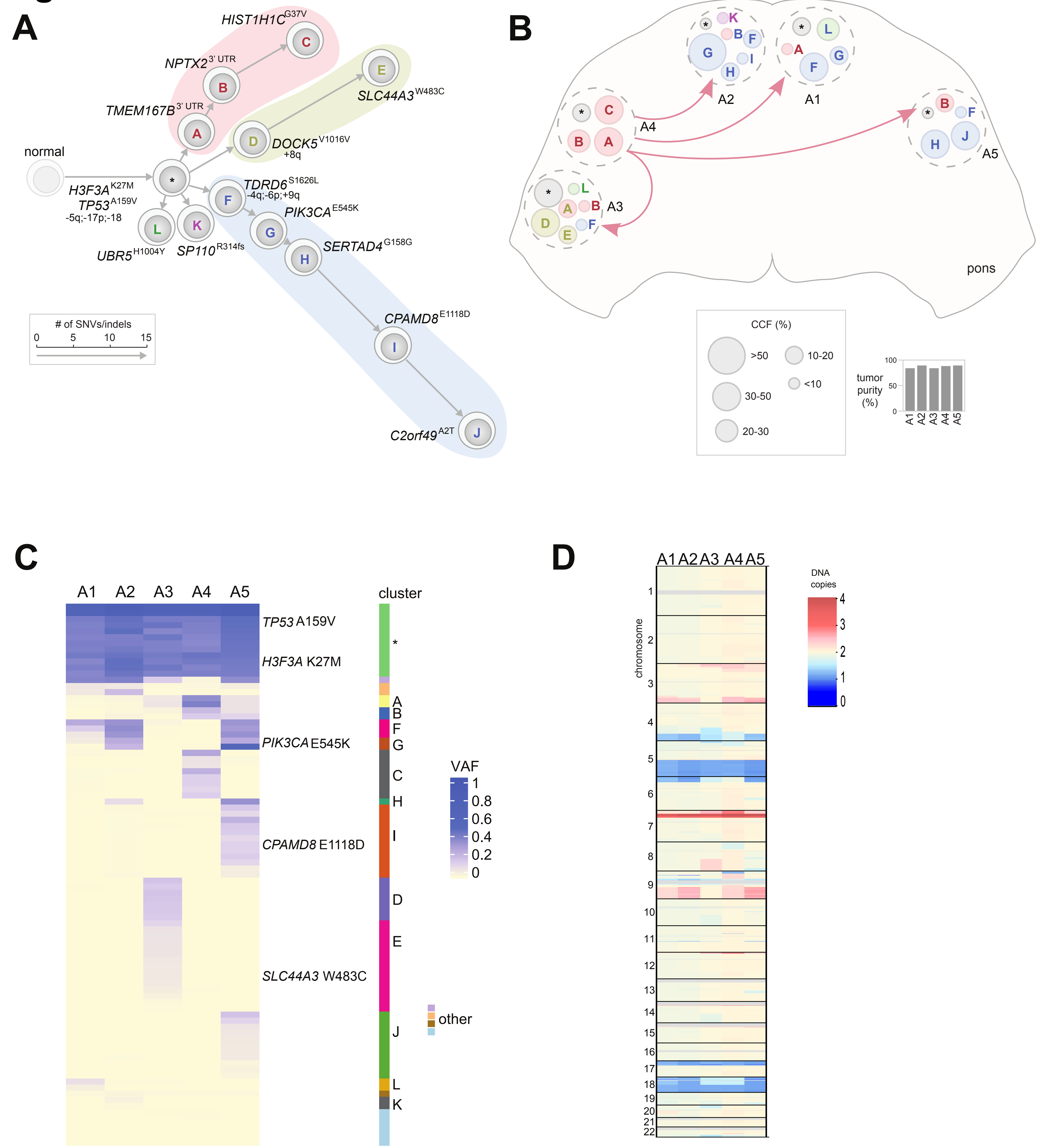

Figure S14: Phylogenetic tree and clonal composition of case 308

(A) The evolutionary tree consists of three long branches (blue, green and pink) and two very short branches(gray). The longest branch (blue) has a PIK3CA^{E545K} mutation and green branch initiated by +8q .(B) Spatial position and clonal composition of 5 profiled tumor regions. Admix of tumor cells from the pink branch present in A1, A2, A3 and A5 likely originated from A4's dominant clone as shown by the pink arrows. (C) Heatmap of somatic SNVs/indels with selected variants marked. (D) Somatic CNV plot of five tumor samples.

Figure S15

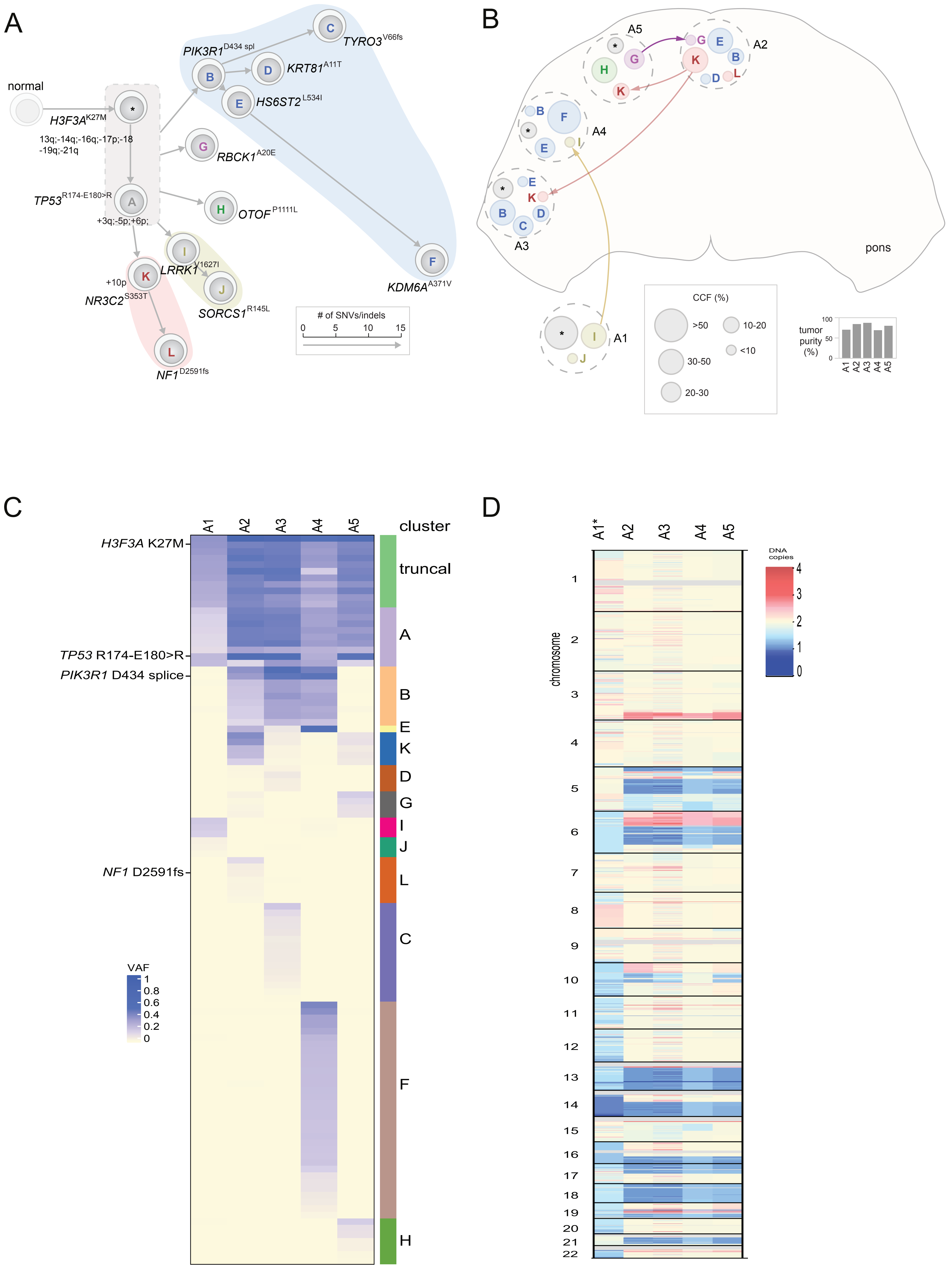

Figure S15: Reciprocal invasion within the pons in patient 324
The two panels (A and B) are drawn using the same style as Figure 3. (A) An evolutionary tree with five major branches. Truncal variants consist of a cluster representing the founder clone (*) and cluster A with varying CCF in different tumor regions which are outlined with a dotted line; the descending nodes can arise either from the founder clone (*) or clone A. (B) Spatial position and clonal composition of 5 profiled tumor regions. The arrows point to potential patterns of tumor cell migration indicating reciprocal invasion between A2 and A5. (C) Clusters of somatic SNVs/indels from the 5 profiled tumor regions. The tumor samples are ordered based on the similarity of clustering pattern. Each cluster is marked by an alphabetic ID and a distinct color at the right. The cluster ID is also used to label the corresponding node on the phylogenetic tree and subclone ID shown. VAF of each mutation is indicated by the blue shade and selective driver mutations are labeled. (D) Somatic CNV plot by SNP array (A2-A5) or exome CNV (A1, marked by *) with blue color indicating copy loss and red indicating copy gain. The tumor samples are displayed in the same order as (D). It should be noted that exome-based CNV in A1 is not included for the summary statistics of Figure 1C. We noted that genome-wide deletion of chr11, 12, 20 and 22 was not supported by allelic imbalance while the BAF of chr3, 5, 6, 10, 21 matches the pattern observed in other samples.

Figure S16

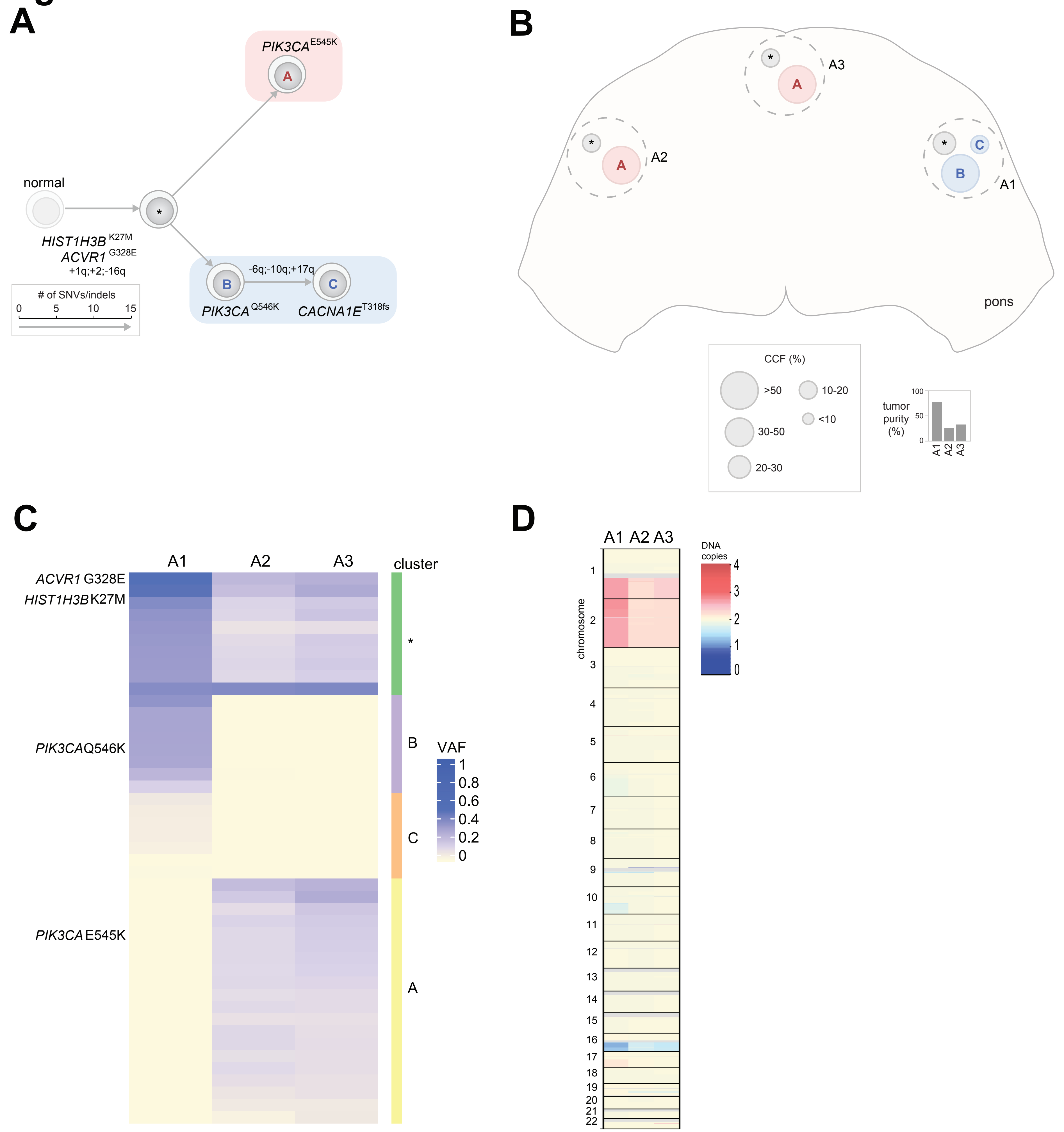

Figure S16:Phylogenetic tree and clonal composition of case 160.
The patient has a H3.1 K27M mutation and *PIK3CA* convergent evolution was found in three different tumor regions. (A) The evolutionary tree consists of two branches, blue and pink, bearing two distinct PIK3CA mutations Q546K and E545K, respectively (B) Spatial position and clonal composition of three profiled tumor regions. (C) Heatmap of somatic SNVs/indels with selected variants marked. (D) Somatic CNV plot of three tumor samples.

Figure S17

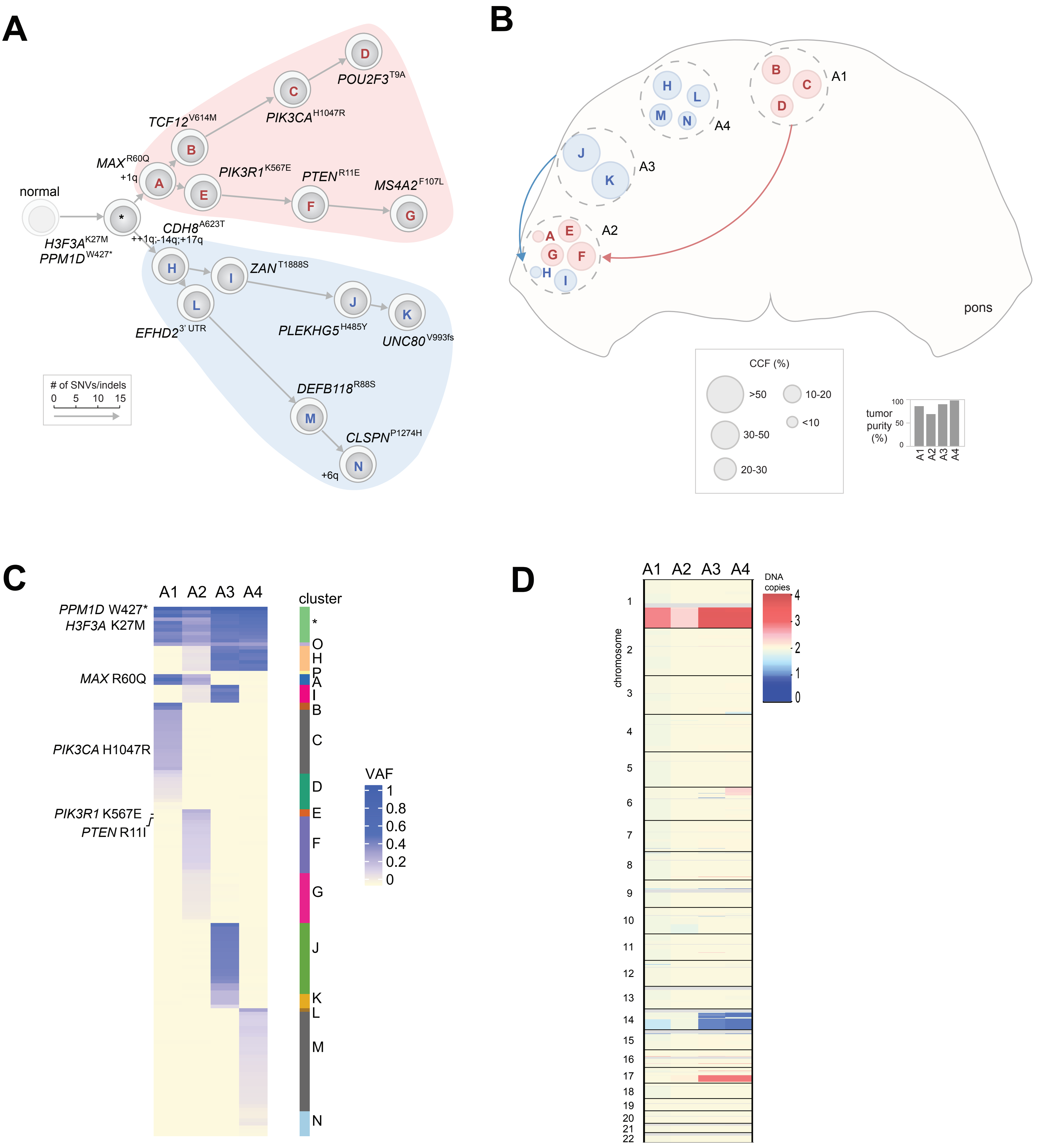

Figure S17: Phylogenetic tree and clonal composition of case 313.
This case has an early clonal divergence accompanied by PI3K convergent evolution. (A) Evolutionary tree has two major branches colored pink and blue. Each branch further bi-furcates with similar lengths between the two branches. The pink branch bears clones with PIK3CA^{H1047R} and PIK3R1^{K567E} mutations while the blue branch is initiated by -14q and +17q. (B) Relative molecular age of the major branches. (C) Spatial positions and clonal composition of each sample. A2 is occupied by tumor cells of the mixed lineages as indicated by the blue and pink arrows. (D) Heatmap of somatic SNVs and indels. (E) Somatic CNV plot of all four tumor samples
